# Students as researchers: What and why seventh‐grade students choose to write when investigating their own research question

**DOI:** 10.1002/sce.21324

**Published:** 2017-12-19

**Authors:** Tuva Bjørkvold, Marte Blikstad‐Balas

**Affiliations:** ^1^ Faculty of Education and International Studies Oslo and Akershus University College of Applied Sciences Oslo Norway; ^2^ Departement of Teacher Education and School Research University of Oslo Oslo Norway

**Keywords:** science literacy, students as researchers, video study, writing

## Abstract

All scientists depend on both reading and writing to do their scientific work. It is of paramount importance to ensure that students have a relevant repertoire of practices they can employ when facing scientific content inside and outside the school context. The present study reports on students in seventh grade acting as researchers. Over an 8‐week collaborative research period, students posed their own research question, attempted to answer it by systematically testing hypotheses, discussing findings, presenting their conclusions, and documenting their process in a written report. Drawing on the perspectives of New Literacy Studies—which sees literacy as socially situated—we analyze the purpose of all the 21 participating students’ texts (*n* = 344). Video observations and interviews with students are used to contextualize the writing events. We find that the students chose to write multiple kinds of texts for a variety of purposes. Analyzing purpose and the context, three stages of socialization into scientific writing is revealed, ranging from what the students write on their own initiative, via texts written through challenges to demanding research tasks scaffolded through writing instructions given by the teacher. Further, the students emphasized the relevance of both the research experience and the writing to their future adult life.

## 1 INTRODUCTION

Texts and socially meaningful ways of using them are essential parts of science (Knorr‐Cetina, 1999; Norris & Phillips, 2003; Osborne, 2007; Sørvik & Mork, 2015). Research has demonstrated that professional researchers and scientists engage in a variety of textual activities not only for documenting their research and presenting it to peers in a number of ways but also for reading, discussing, and commenting on others’ research. Put simply, scientists depend on literacy practices in performing their scientific work (Lemke, 1990; Moje, Collazo, Carrillo, & Marx, 2001; Norris & Phillips, 2003; Yore et al., 2004). As emphasized by Howes, Lim, and Campos (2009), because reading, writing, and speaking play foundational roles in professional scientists’ work, literacy practices should be central to science teaching.

There has been a shift in different national curricula in the past decade, and several countries are striving to increase their educational systems’ emphasis on the role of language (Australian Curriculum, 2016; Department of Education Norway, 2013; National Governor's Association Center for Best Practices and Council of Chief State School Officers 2010). Still, while the literacy practices associated with science are multifaceted and complex, those associated with school and school science are often narrow and revolve around reproducing scientific facts and memorizing information (Danielsson, 2010; Osborne, 2007). In a review of the role of text in school science, Sørvik (2015, p. 19) concluded that this role is often “characterized by a dominant (but unutilized) use of the science textbook, coupled with reading/writing activities that appear to be embedded in a transmissive mode of science teaching.” In a comparison of high school students from Sweden, England, and Australia, Lyons (2006) found that students reported remarkably similar experiences of school science, suggesting a transmissive pedagogy and decontextualized content. Further, the international Trends in International Mathematics and Science Study (TIMSS) found that science textbooks play a key role in school science instruction in both primary and secondary schools across countries (Martin, Mullis, Foy, & Stanco, 2012). To summarize, a large body of research suggests that literacy practices in school science are predictable and somewhat narrow, even though there is a trend in several countries to change the curricula to enhance science literacy. There appears to be a significant gap between textbook‐based literacy practices, where science is often taught as undisputed fact (Osborne, 2007), on the one hand, and the literacy practices encompassed by scientific practice on the other hand.

According to Norris and Phillips’ (2003) seminal reconceptualization of scientific literacy, the fundamental sense of scientific literacy involves fluency in the language, discourse patterns, and communication systems of science, whereas the derived sense of scientific literacy is about being knowledgeable, learned, and educated in science. If we concern ourselves with only the derived sense of scientific literacy, we might be ignoring “crucial aspects of the substantive, epistemic, and social dimensions of science and scientific literacy” (Sørvik, Blikstad‐Balas, & Ødegaard, 2015, p. 41). How and why students engage with texts matter because it is crucial that students develop a variety of critical literacy practices and explore questions with no clear, factual answers available in a textbook; such exploration resembles practices outside school, and such exposure is therefore also important to experience as a student. The types of discursive experiences and literacy practices in which students actually engage and the manner in which these relate to the overall social literacy practices associated with science and research are of paramount importance if we want students to have a relevant repertoire of practices to employ when facing scientific content inside and outside of the school context. As Osborne (2007, p. 179) argues, students must have opportunities to develop the ability to think critically about scientific evidence. To do so, it is necessary for them to be faced with data that lacks a clear interpretation, and they must be given occasion to consider plural alternatives and explanations (Osborne, 2007). Unfortunately, the previously mentioned studies indicate that this might not be the case in many classrooms.

In this article, the authors report on a classroom study in which seventh‐grade students from a Norwegian elementary school were given the opportunity to act as researchers through participation in a national research contest. The participating students were encouraged to design their own research project, pursue their research hypotheses with data of their choice, and interpret and discuss their findings before presenting the whole process and the scientific outcome in a final written report. This report was then evaluated by a jury composed of readers outside the school setting. The students spent 8 weeks working on a self‐determined research question, namely, “How far away can dogs smell a treat?,” and hypotheses concerning the color of the dog, moisture of the treats, size of the dog's nose, and training, size, and breed of the dog. This process also included conducting actual experiments with dogs from different breeds and sizes. This way of working captures many of the elements associated with best practice in science education: student autonomy, an inquiry‐based approach to scientific data, and authentic audience outside a school setting.

Our study provides access to a unique case that has been thoroughly documented. First, it represents an unusual opportunity to investigate the writing practices associated with students’ research processes, as opposed to those associated with engagement in more traditional tasks related to school science. As Howes et al. (2009) argued, it is crucial to encourage and aid children to use evidence from the natural world to address their own questions. This case study documents the working processes of students who were given such opportunities. Second, the study sheds light on writing practices that produce texts of which the teacher is not the only intended reader; the absence of this element has been a critique of school literacy in general and school science in particular (Af Geijerstam, 2006; Barton, 2007). Because students’ engagement in authentic writing activities in school science is linked to increased comprehension and production of both informational and procedural science texts (Purcell‐Gates, Duke, & Martineau, 2007), it is important to investigate situations in which students actually work with authentic writing events. Here, authentic writing events are understood as events where the purpose reflects the purposes of writing outside the school context. For instance, writing a report to present and discuss actual findings is considered a more authentic writing event than writing a report primarily to practice writing in the report genre. Furthermore, the event serves the socially communicative purpose of informing (Purcell‐Gates et al., 2007, p. 14), which is an important and understudied purpose in school science.

## 2 THEORETICAL PERSPECTIVES: WRITING AS A SOCIAL PRACTICE

To analyze how and why students as researchers write, we draw on the notion that literacy is a social practice involving texts (Barton, 2007; Gee, 2008; Gee, 2015), which is a central perspective of New Literacy Studies (hereafter, NLS^1^). A sociocultural approach to literacy implies that all meaning is situated in specific practices and experiences (Gee, 2000). Literacy is no static concept. It is not a set of skills or competences people *have obtained* or *will attain* but a reference to something people *do*. Thus, writing cannot be detached from the social settings in which it is embedded, because it requires scaffolded socialization to learn to handle a given text in a given way (Gee, 2001).

Understanding how and why texts are written and used is essential to understanding literacy and the practices texts generate, enable, and maintain. Historically, emphasis on the actual use of texts in science education has been limited, but a broader view on literacy and a concern for students’ literacy practices is emerging in contemporary research (Blikstad‐Balas & Sørvik, 2014; Howes et al., 2009; Norris & Phillips, 2003; Osborne, 2002, 2007; Sørvik et al. 2015; Sørvik & Mork, 2015). Sørvik and Mork (2015, p. 276) explained how a social view of literacy “provides science education with the theoretical perspectives to examine the role of literacy in a transcending science subject.” As they emphasized, a theoretical perception of reading and writing as situated within a social context, enables researchers to “consider how literacy is a part of contexts that influence science education and are relevant to the long‐term goal of scientific literacy” (Sørvik & Mork, 2015, p. 276).

In the present study, we are concerned with what and why students write when conducting their own research project. We employ two terms derived from NLS to investigate students’ writing, namely, *writing event* and *writing practice*. These are derived from the terms *literacy event* and *literacy practice* Barton & Hamilton, 2000, and their use signals that our main focus is on writing rather than other aspects of literacy, such as reading and orality. A *writing event* can be defined as any occasion in daily life in which writing plays a role (Hermansson, 2011; Rish, 2015). Writing events, then, are empirically observable activities in which writing is undertaken. Writing includes production of all kinds of texts, both handwritten and digital, and texts in which the written word is combined with other modalities such as drawings, figures, and tables. We argue that a broad definition of writing, one that goes beyond written text, is particularly important from a science education perspective, as scientific knowledge often depends on a variety of modalities. With reference to Latour (1986) and Prain and Tytler (2013, p. 2), we emphasize how scientific thought depends on representational tools and how representational work plays a foundational role in interpreting, representing, and assessing scientific claims. Science cannot be communicated by verbal language alone, but is rather dependent on several modes of communication (Lemke, 1998). Knain's (2015) work on multimodal representations in science shows how science is a particularly good example of a strongly multimodal discourse. In school science, “forms of representations are not only learning goals, but also means for learning” (Knain, 2015, p. 84).

As argued by Barton and Lee (2013), a text makes a good starting point for analysis because it can act as a fixed point of interaction. Research framed in NLS is typically not concerned with the analysis of texts per se; rather, the focus is on what people do with texts and their purposes for engaging in, for instance, writing (Barton & Hamilton, 1998). *Writing practices* constitute a specific type of social practice, namely, social practices involving writing (Hermansson, 2011). In line with Street's (1995) definition of the term literacy practice, we use the term writing practice to refer to the behavioral, social, and cultural conceptualizations that give meaning to the uses of writing. Similar to any other social practice, a writing practice is not observable. Events are the visible tip of the iceberg, and through the study of these observable units, one can infer practices (Hamilton, 2000). An important methodological perspective in NLS is that rather than simply asking participants what they “do with texts,” we should rigorously look for patterns in participants’ writing events as they occur (Blikstad‐Balas, 2013). In the present study, this is achieved by analyzing the texts written by the students at different stages in their research process and by considering contextualizing data from the interviews with the students about their writing events and the recordings of the writing events.

## 3 LITERATURE ON STUDENTS’ WRITING IN SCHOOL SCIENCE

In the following review, we focus on two areas of particular interest to the present study, namely, research on writing in school science and research about students as researchers.

As noted earlier, a large body of literature identifies the general literacy practices associated with school science as somewhat narrow and revolving around reproducing scientific facts and memorizing information (Danielsson, 2010; Lyons, 2006; Osborne, 2002, 2007; Sørvik, 2015; Sørvik & Mork, 2015). Not surprisingly, similar results were found in detailed investigations of what characterizes students’ writing in school science. For instance, Zangori and Forbes (2014) investigated how third‐grade students wrote scientific explanations. One of their key findings was that across the three classrooms studied, a significant majority of writing samples from the students did not include any facet of scientific explanation. Furthermore, the results of their qualitative analysis of these student writing samples identified the students’ writing as largely defined by data description without discussion of cause, effect, or mechanism. Similarly, Af Geijerstam (2006) investigated writing in school science in Grades 5 and 8 in Sweden. Her study was based on texts written by 97 students, interviews about these texts, and classroom observations. An important finding of this study was that students found it difficult to talk about their own scientific texts in terms of both articulating the main ideas in their texts and discussing the texts’ function(s) and potential readers.

Two main approaches have been employed to integrate writing into the teaching and learning of science.^2^ In the first approach, often referred to as the genrist viewpoint (Prain & Hand, 2016) or the “learning to write” perspective (Sampson, Enderle, Grooms, & Witte, 2013), it is considered essential to make available to students the language conventions of science and familiarize them with the representational systems needed to demonstrate scientific literacy. Thus, this approach focuses on teaching students subject‐specific writing skills identified as central in scientific literacy practices. This approach aims to provide students with the writing conventions and writing skills required to be able to write well in science (Alley, 1996; Halliday & Martin, 1993; Indrisano & Paratore, 2005; Veel, 1997). The second perspective, often referred to as emphasizes that to acquire scientific literacy, students should use writing to develop better understanding of content and that students must experience firsthand how writing can be done in different ways for different audiences. An essential part of this approach, then, is that composition can help students in their processes of understanding and reasoning about a subject, in this case, school science (Klein, Arcon, & Baker, 2016; Levin & Wagner, 2006; Prain & Hand, 2016; Rowell, 1997; Sampson et al., 2013).

The writing‐to‐learn perspective fits well with the present study because it underscores the need for realistic writing practices in school science and focuses on both the writing process and the content. Within the writing‐to‐learn perspective, one can focus on both epistemic learning, as presented above, and learning as socialization (Klein & Boscolo, 2016). Learning through writing as socialization has several aspects, as it would for emerging professionals, writing genres associated with a discipline, or apprentices in writing as professionals (Carter, Ferzli, & Wiebe, 2007). Recent trends in the research on writing as a learning activity show that other social aspects of writing have also been foregrounded lately, such as collaboration and social support for writing (Klein & Boscolo, 2016). This latter understanding of writing to learn as socialization is crucial in the present study, as we investigate students who are conducting their own research inquiries and writing for a number of purposes. The student writing examined in this study took place as part of a science competition, explained in more detail below, of which one of the main goals of the science competition in which the students in this study take part is to provide students with experience with research methods and ways to work as a researcher. This aligns well with the writing‐to‐learn perspective.

Although interventions connected to the integration of science and writing in elementary school have been researched from several angles, often in quasi‐experimental studies (Cervetti, Barber, Dorph, Pearson, & Goldschmidt, 2012; Chen, Hand, & McDowell, 2013; Ødegaard, Haug, Mork, & Sørvik, 2014; Peck, 2010; Sampson et al., 2013), there are fewer studies of “naturally occurring” lessons, where science and writing are combined systematically, as is the case in our study. Larson and Marsh (2005) discussed a classroom where inquiry and writing are integrated by working thematically and systematically over time. In that study, students used writing when needed in their work, for instance, to produce a guide to a local park, with visitors as the intended readers. The work appears to be teacher‐led but is placed in a natural setting. This is also the case in another study, in which fifth‐grade students wrote online arguments in a closed learning environment (Choi, Hand, & Norton‐Meier, 2014). The students’ discussions were part of an argument‐based inquiry approach used in‐class, but the online discussions served to enhance students’ reflections. Another study was designed to create an environment in the sixth grade to promote spontaneous writing of notes during inquiry (Garcia‐Mila, Andersen, & Rojo, 2011). Although the study was designed in advance, the writing aspect was not planned in detail by the facilitators, and the study shows that students can take notes of their own accord, especially for memorization and organization.

## 4 STUDENTS AS RESEARCHERS

Engaging students in research is a methodology in science education (Elmesky & Tobin, 2005) and philosophy (Mills, O'Keefe, Hass, & Johnson, 2014) that involves *doing what scientists do* within science education (Howes et al., 2009, p. 190), in other words, engaging students in the practices of science (National Research Council, 2012, p. 42). Research on students as researchers can be categorized into the following three main approaches, of which the first is the one relevant to our study.

The notion of students as researchers is emphasized differently among different scholars. First, research is seen as an essential part of science, fundamental to how we establish new knowledge and develop our understanding of the world (Norris & Phillips, 2003). Because research is central to science, an essential part of science education is to teach students about the research process, often referred to as inquiry or epistemological understanding of the nature of science (Abd‐El‐Khalick et al., 2004). This strategy allows students to act as researchers within the subject of school science. Various terms are used to refer to the phenomenon of students conducting research: *students as researchers* (Elmesky & Tobin, 2005), *kids/children as researchers* (Dahl, 2014; Mills et al., 2014), or the broad term *(open) inquiry* (Colburn, 2000; Howes et al., 2009). These differently labeled approaches have two important aspects in common: First, students pose their own research question, investigate it systematically, collect data, and compare findings with their own ideas or initial hypotheses (Dahl, 2014; Howes et al., 2009). Second, students share their research, either through writing or orally, with an audience (Elmesky & Tobin, 2005). In the present study, we use the term *students as researchers* to emphasize our investigation of the practices of students within the school and the crucial aspect of students researching their own research question.

The second way to consider students as researchers is as apprentices or research assistants in studies conducted by adults (Elmesky & Tobin, 2005; Mason & Watson, 2014). As Elmesky and Tobin (2005) argued, involving students in the research of an adult team of experts in the field, namely, urban school science education, can be advantageous. Students can help target research questions, formulations, and methods aimed at groups of informants with whom they are familiar (Dahl, 2014). Moreover, data collection by children can provide valuable insight that adults may miss owing to differences in age, power, or background (Dahl, 2014).

The third way to highlight students as researchers involves seeing the child as a unique actor and voice in the research community. As Dahl (2014) pointed out, children are the only ones who know what their childhood is like, and therefore, they should be given the opportunity to tell their stories. However, adults control the research results being published, and the children's voices as researchers might be more of an ideal or norm, especially in challenging situations (Mills et al., 2014).

The present study concerns students conducting open inquiry, which is a way of working that is debated partly because the learning outcome is not always clear. Kirschner, Sweller, and Clark (2006) criticize the approach for not working because of minimal guidance. Hattie's (2009) meta‐analysis shows mediocre results for inquiry. Furthermore, a synthesis of 138 inquiry studies indicates that high levels of inquiry in instruction are not associated with positive learning outcomes for students (Minner, Levy, & Century, 2010). Another study concerning openness in inquiry connected to the Programme for International Student Assessment (PISA) found that the highest level of inquiry, defined as students asking their own questions, resulted in the lowest level of science achievements for the students (Jiang & McComas, 2015).

However, it has also been found that open inquiry gives students increased communication skills, increased participation in authentic science through inquiry, and ownership of their work (Hubber, Darby, & Tytler, 2010). Zion and Mendelovici (2012) regard open inquiry as the highest level of inquiry and, therefore, argue that it is something students should experience. The Next Generation Science Standards foregrounds students’ activity in science inquiry: “As in all inquiry‐based approaches to science teaching, our expectation is that students will themselves engage in the practices and not merely learn about them secondhand” (National Research Council, 2012, p. 12). The standard expects engagement through inquiry, and this might lead to other learning outcomes than the ones tested in the lager studies cited, for example, learning in writing and socialization into science through writing.

## 5 PRESENT STUDY

The main aim of the present study is to investigate the writing practices of students conducting a research project. To do so, we deliberately chose to follow what we believe to be an atypical or deviant case (Flyvbjerg, 2006), in the sense that the participating students were provided with an opportunity to conduct in‐depth research over an extended period (8 weeks). As the reviewed literature suggests, this is not typical in school science. However, as our literature review also suggests, several scholars within the field of science education emphasize the importance of providing students with authentic writing activities and the importance of encouraging students to interpret scientific evidence by working with data that lack clear interpretations (Osborne, 2007; Purcell‐Gates et al., 2007). Thus, while the chosen case may not be typical for school science instruction in general, but through it, we attempt to answer the call to bridge the writing practices of school science and science as a discipline.

The present study investigates what and why students as researchers choose to write. It addresses the following main research questions:

1. What is the purpose of the students’ writing as they are conducting research?
2. What characterizes the initiation of texts when students are conducting research?

While the first question is concerned with the totality of texts in the students’ research project and identifying the purpose of their writing, the second question addresses the fact that students in school science typically write because they are told to, thus making it interesting and important to investigate the writing events in which students as researchers themselves decide to write as an integral part of their research process. The two questions are interrelated in the sense that we intended to investigate the actual texts produced by students and the roles of those texts in the research project (1), as well as the students’ reasons for writing (e.g., whether they write to remember, plan experiments, documents their findings, or present their research to others). Furthermore, to get an understanding of the writing practices from which these writing events arise, a last research question is posed:

3. How do students perceive their writing during their work as researchers?

## 6 METHODS

### 6.1 Participants and context

The students in this case study participated in a nationwide research competition called “The Nysgjerrigper Science Knowledge Project,” organized by The Research Council of Norway (2016). In Norwegian, the term “nysgjerrigper” describes an individual who asks questions about everything imaginable, an equivalent to the English term “curious George.” The term refers to both boys and girls, and although this research encompasses more boys than girls (because there were more boys than girls in the class participating in our study), just as many girls as boys participate in the Nysgjerrigper Science Knowledge Project.

In this competition, children from elementary school, Grades 1–7, are invited to carry out small‐scale research projects and compete for the annual Nysgjerrigper Science Award. Participants are encouraged to identify research questions and hypotheses, plan projects, gather data, draw conclusions, and communicate the results of their research to others in a written report. The reports are then judged by a jury of researchers (not teachers), and winners are announced. Since the competition was established in 1990, about 2,500 students have participated annually (Nysgjerrigper/RCN, 2013). The research competition is organized outside of school, and schools can choose to participate. The Norwegian national curriculum has two subjects (natural sciences and social sciences) in which research methods are a consistent topic from Grades 1 to 11 (Ministry of Education and Research, 2013a); thus, student participation in the competition fits the needs of Norwegian schools well.

The case in the present study is purposely chosen because its specific context, students participating in the research contest, was the starting point of our study. The chosen class consisted of six girls and fifteen boys (21 students in total) in the seventh grade (age 12), the last year of elementary school in Norway. The class is not particularly high achieving, for instance, in terms of national test scores, but it should be noted that the class and the teacher were well accustomed to the research contest because they have participated several times in the past. Participation in this particular science contest has been an explicit part of the locally adapted curriculum of this school for years. This means that the studied case represents normal classroom practice at the school, although participation in the competition is not that common across Norway.

The entire research period lasted 8 weeks; the period was naturally demarcated, beginning with finding a research question and ending with submitting the report (Stake, 1995). Normally, the class would work on their interdisciplinary project for one to two 60‐minute lessons per day, with around five to seven lessons per week. The student groupings varied. Most often, they organized themselves according to interest and ability in groups of two to eight students. On other occasions, the teacher divided the entire class into similarly sized groups.

Initially, the students came up with 110 possible research questions, which in turn were reduced to five “researchable” research questions. Then, the class discussed the five options and by vote, they decided to investigate the research question “How far away can dogs smell a treat?” This became the shared research question. Different groups identified methods to test their hypotheses, which concerned dog breed, size of the dog's nose, what kind of training the dog had, the moisture content of the treat, and the color of the dog's fur, among other criteria. The students gathered empirical data by conducting dog experiments and interviewing experts such as veterinarians, a pet shop owner, and a police officer who worked with a narcotic detection dog. The data collected was analyzed in light of the hypotheses; further investigations were undertaken when needed. Throughout the 8‐week period, the students wrote various texts supporting their research, and the whole process ended in one common written report, submitted from the class as a research team. The entire research process was highly collaborative.

The teacher was the overarching project facilitator. She ensured that the students’ research process moved forward by following up with the students from the time the research question was established until the report was complete. Usually, lessons would start out with a plenary session (15–20 minutes) led by the teacher, during which the current state of the project and future challenges faced by the class were discussed. After this session, the students would organize themselves into groups in different locations, and the teacher would supervise the different groups while being available to answer questions. Mainly, she would help out with practical issues such as obtaining the specific equipment required by the students or following up on the challenges reported by the students. On most occasions, the teacher asked questions about project progress and possible obstacles. The teacher would rarely comment directly on a text; rather, she would question the purpose of a given text and what the student wanted to achieve through writing.

The class participating in the present study had been socialized into doing their own research over the course of their six previous years at the same school, with different teachers. They were accustomed to working collaboratively with one research question and submitting a common written report. As the annual period of inquiry usually lasted 4–8 weeks, the rest of the school year had a different kind of organization and curricular approach, including textbooks in every specific subject, as opposed to interdisciplinary instruction, and teacher‐assigned homework.

### 6.2 Research design and data collection

The empirical data in the present study was collected by the first author during a period of 8 weeks in 2014. It encompasses a collection of texts written by the students, video observations, and interviews with the students, as described in Table 1.

**Table 1:** Empirical data

Primary data sources	Number
Student texts	344

Secondary data sources	
Video observation	26 lessons, total of 104 hours
Interviews, students	22 interviews (161 minutes total)—20 students in different groupings were interviewed at different stages during the 8 weeks

### 6.3 Texts

In the present study, data were collected primarily from the 344 student texts produced during the period over which the students worked on their research project. Both digital and analog texts are included. The texts were collected during breaks after each lesson, either as copies of digital files or scans of handwritten texts. The students retained their originals at all times. No one refused to have their text scanned, although all students were informed of this option and knew they could withdraw their consent to participate at any time.

Our definition of texts is broad and included in addition to prose multimodal texts, drawings, and still and moving pictures. The multimodal texts consisted of at least two modalities, such as prose, drawings, pictures, tables, charts, and bulleted or numbered lists. To define what counted as a mode, we examined each text to determine what functioned to convey meaning in the material (Bezemer & Kress, 2008). For instance, single words written in bold in prose were seen as a mode in an instruction for the dog experiments, as it functioned to enhance important features. We have not conducted multimodal analyses of the texts, but we emphasize that the whole multimodal text has been used when categorizing a given text's function in our analyses.

As pointed out by Barton and Lee (2013), texts can no longer be seen as stable entities, and it is sometimes challenging to say where a text starts and ends. A text was labeled and counted as a new text if other students joined the writing or if major changes were made to an existing text. A major change is, for instance, changing the format from running notes to a table, merging the contents of several texts into one, or altering the organization of a text. A text is counted as one single text regardless of how many authors it has. Therefore, some of the texts in our material have only one or two authors, whereas others may have as many as eight authors.

The texts were written in Norwegian. Thus, all extracts have been translated by Author 1 and proofread by a peer. This is also the case for quotes from video data and interviews.

A key methodological challenge when collecting artifacts (such as student texts) from the classroom is that the artifact itself may reveal little about how and why it has been used and about instructional interactions between teachers and students (Martínez, Borko, & Stecher, 2012). However, we attempted to counter this limitation by including contextualizing data. As elaborated later in the paper, the texts are seen in context because we video‐recorded the students during their research process and interviewed them while the research process was still ongoing.

As the study is a case study, and all texts are collected within the same period of research by the students, we were not able to investigate students’ development or compare the writing practices to students’ practices prior to or after the research project period. Measuring gains in disciplinary writing is not among the aims of the present study; rather, the main aim is to explore the purposes for which students write during their research process.

### 6.4 Video data

Video studies have been essential in providing new insights into the detailed and multifaceted activities occurring in classrooms (Derry et al., 2010; Goldman, Pea, Barron, & Derry, 2007; Klette, 2009). In the present study, video observations were made using two different types of cameras, fixed and head‐mounted,^3^ with simultaneous focus on the overall activities of the class and specific activities of selected students.

**Figure 1:**
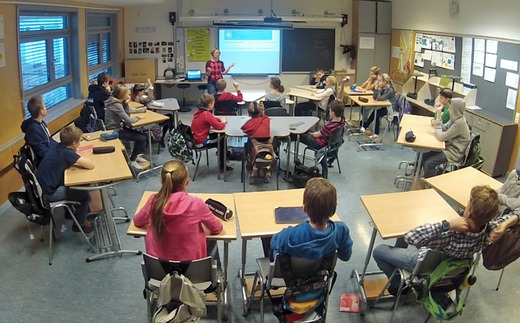
Capture from whole‐class camera. Teacher leading class discussion [Color figure can be viewed at wileyonlinelibrary.com]

The fixed camera was located in the back of the classroom to capture the activities of the teacher and the class in plenary settings (Figure 1). A wireless microphone was placed on the teacher. Together, the built‐in microphone in the fixed camera and the microphone on the teacher captured both the teacher's words and the plenary discussions in the classroom. The camera recorded all activity in the classroom, but as the teacher moved between groups in different rooms during group work, not all sound was recorded.

**Figure 2:**
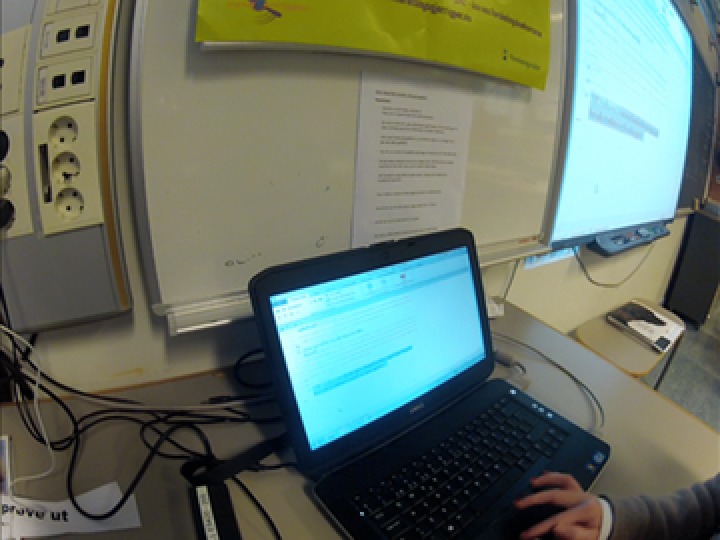
Capture from head‐mounted camera. Student writing form for interviews [Color figure can be viewed at wileyonlinelibrary.com]

**Figure 3:**
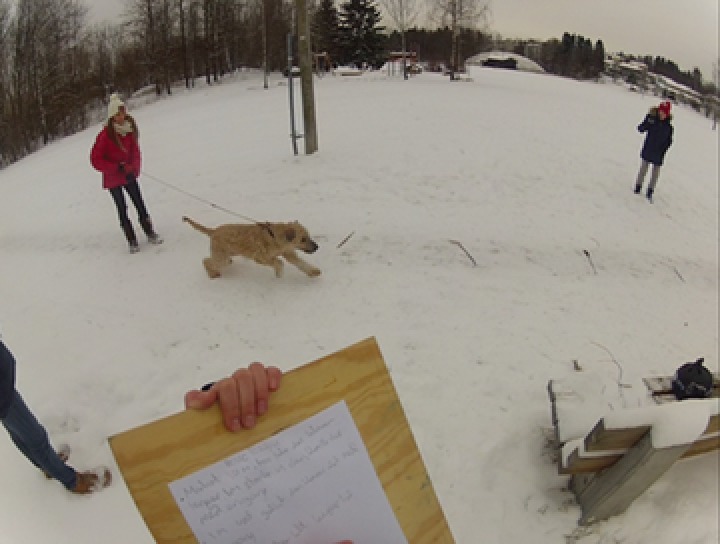
Capture from head‐mounted camera. Student writing notes from dog experiment [Color figure can be viewed at wileyonlinelibrary.com]

**Figure 4:**
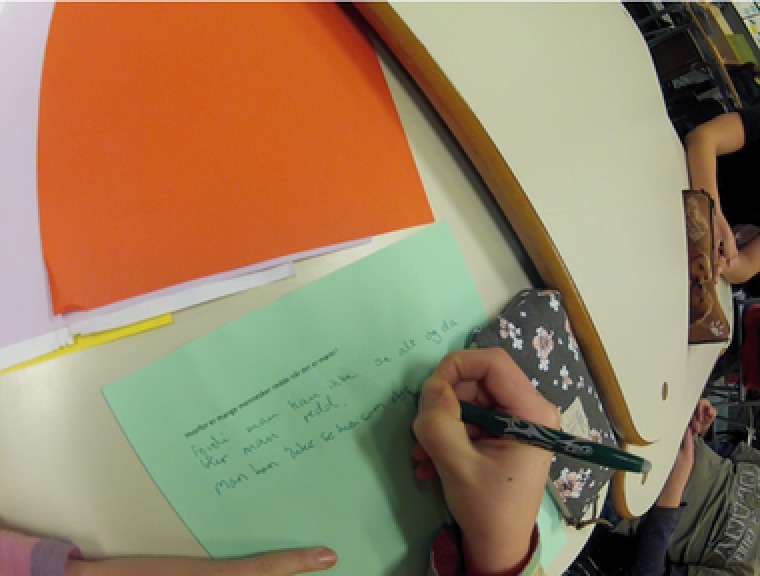
Capture from head‐mounted camera. Student writing thoughts about possible research question [Color figure can be viewed at wileyonlinelibrary.com]

**Figure 5:**
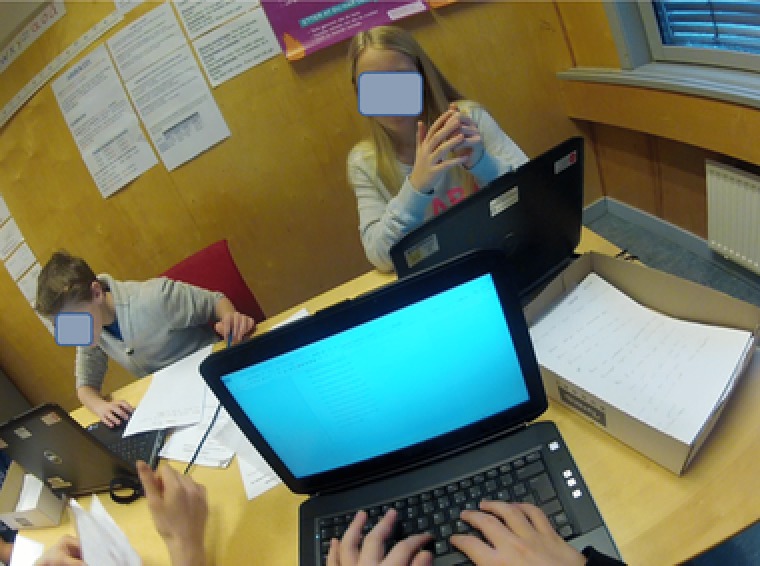
Capture from head‐mounted camera. Student working in group [Color figure can be viewed at wileyonlinelibrary.com]

**Figure 6:**
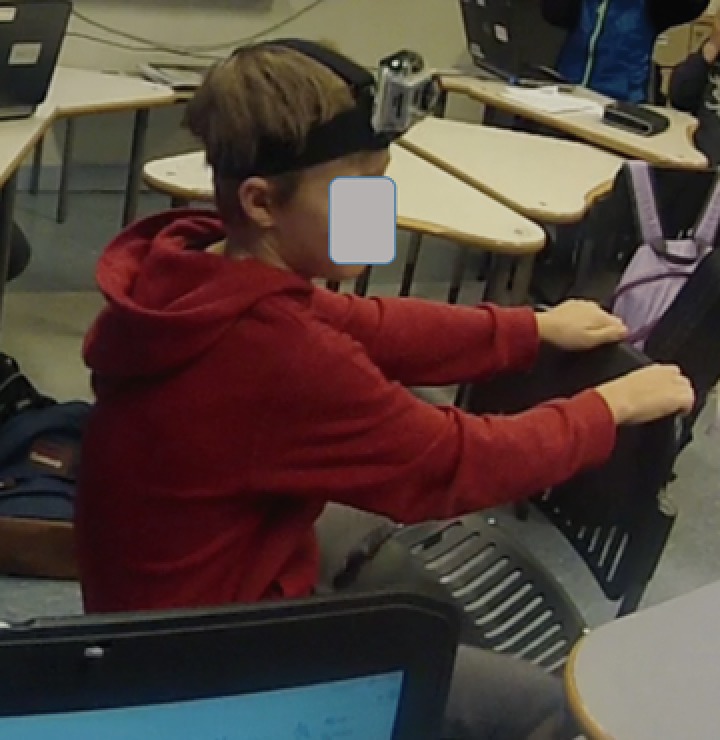
Student with head‐mounted camera [Color figure can be viewed at wileyonlinelibrary.com]

To obtain detailed insight into the students’ activities, three head‐mounted cameras were also used. This way of gathering data has proven useful in several research projects investigating texts in use (Bjørkvold, 2015; Blikstad‐Balas, 2012; Blikstad‐Balas & Sørvik, 2014; Maltese, Danish, Bouldin, Harsh, & Bryan, 2016). In the present study, video data are used to provide context for the primary data sources, namely, the collected texts written by the students. To obtain a broad representation of the work in the class, 19 of the 21 students wore the head‐mounted cameras by turn. There were never two cameras in one group of students, and the same student kept the camera throughout the day. The head‐mounted cameras were used for two main purposes: to obtain a broader picture of the writing events revolving around the initiation of writing and to act as stimulation in student interviews (Figures 2, 3, 4, 5). The figures give an impression of the kind of data this design provided, and how the camera looks on a student (Figure 6).

### 6.5 Interview data

Video‐stimulated interviews create the opportunity to obtain the participants’ view of an observation, thereby possibly enhancing the reliability of the data (Guba & Lincoln, 2007). Video‐ and/or text‐stimulated interviews were conducted to gain a richer understanding of the writing events. Video clips of these writing events, recorded by the head‐mounted cameras, were shown to the student who wore the camera and the students he or she had collaborated with, typically around three students. The clips, each lasting 1–3 minutes, were presented no more than 60 minutes after an event had taken place, usually after a break. Through the video‐stimulated interviews, Author 1, who conducted all the interviews, aimed to get the students’ own views of the writing events and their understanding of their situation in their own words. Therefore, the students were asked to elaborate on what was going on in the video clips, and the interviewer followed up on themes that arose, focusing on why choices were made. Furthermore, the interviews followed an interview guide that highlighted the initiation of writing, purpose of writing, and significance of the writing event, resulting in semistructured interviews (Kvale & Brinkmann, 2009). Altogether 22 interviews were conducted, with an average length of 7:30 minutes and ranging from 1:17 minutes to 14:03 minutes. The interview group size varied from one to eight students, with the typical size being three students. Overall, 20 of the 21 students in the class were interviewed about different texts they had produced during the 8 weeks.

## 7 DATA ANALYSIS

The main aim of our analysis was to get an overview of all the texts and writing events in which the students engaged and to gain insight into their writing practices. The analysis was performed in four steps. First, the first author identified all texts collected during the 8 weeks over which the students conducted their own research. The texts were labeled using their titles/headings or with a short description if no heading was used by the students. Drawing a line between different texts and different writing events is a common challenge for literacy researchers (Barton & Lee, 2013; Blikstad‐Balas, 2013; Hamilton, 2000) in general and collecting texts during a collaborative research process with 21 participants engaging in a large number of writing events does not diminish this challenge. Thus, the first author identified the texts that should “count” as a unique text and those that should be considered as different versions of the same text (and hence not be registered as a new text), as described on page 311. In the present study, 344 texts were identified, and these texts constitute the main source of data in the present study.

Second, using open coding, which involves searching the data broadly for relevant instances (Cohen, Manion, & Morrison, 2011), the initial phases of the writing events connected to each of the texts were identified. This was done by repeatedly viewing the video data connected to the registered texts. As previously mentioned, it is challenging to identify writing events in a complex classroom situation. Therefore, the identified text was the starting point of the analysis; thereafter, video‐interview data were included for contextualization.

In the third phase, selective coding was conducted (Creswell, 1998) to identify a core around which the codes presented in the second phase revolved. This was done according to both the texts and the writing events. The codes were mutually exclusive (see Tables 2 and 3 for an overview of the categories).

**Table 2:** Categorization of texts according to function

Category	Definition	Example
Thinking text	Text used to clarify own thoughts, introvert	Log, mind map
Working text	Text used to gather information, extrovert, not meant to present	E‐mail, request
Presentation text	Text used to present final product, extrovert	Article, table of results

**Table 3:** Categorization of texts according to initiative

Category	Definition	Example
Writing instruction	The teacher explicitly instructs the students to write	“Make an explanatory drawing about the dog's sense of smell”
Open challenge	The teacher gives the students a challenge to solve, presenting purpose of the challenge and, often, the intended recipients of the information, but no guidance on how to proceed; thus, students may choose to solve the open challenge by writing	“How can we get hold of dogs to do research on?” (this open challenge was solved by written letters to dog owners asking for permission to use their dogs)
Student initiative	The students start writing without prior encouragement from the teacher	Notes from experiments

Finally, the interview data were analyzed through *meaning condensation*, which involves condensing informants’ statements into shorter formulations (Kvale & Brinkmann, 2009) to gain a fuller understanding of the writing events; this was done using QSR NVivo 10 software.^4^ Doing so provided insight into the features triggering the students’ writing; such data may, at times, not be visible on video, for instance, if a decision was made without visible or audible planning. By talking to the students, we explored the students’ own conceptualization of writing (Barton, 2007).

### 7.1 Analytical framework

Two approaches were used to get an overview of the students’ texts. First, we asked what purpose the text served in the research process. The second, and main, approach was determining why the text was initially written.

#### 7.1.1 What purpose does the text serve?

First, the texts were sorted according to purpose, building on the theory of Hoel (1997) and a previously developed concept of Bjørkvold (2013). The main idea in this framework is that texts serve different purposes. In this study, the purpose of writing is operationalized by three categories, namely, “thinking texts,” “working texts,” and “presentation texts,” based on the main function they serve for the author at the time they are written. Needless to say, all writing requires thought and work, and all writing can also be seen as a (re)presentation. However, this framework and these labels were chosen to highlight the starting phase of writing, not to cover all the purposes a text can serve at different stages of the process. Our understanding is therefore narrower, for instance, than the one defined in the wheel of writing as *knowledge development* and *exchange of information* (Matre & Solheim, 2015) or understood in context as the intention behind an action (Halliday & Matthiessen, 2004).

We wanted to categorize texts depending on their main function within the research process, although texts may serve multiple purposes simultaneously. This choice should be seen in relation to the large body of research suggesting that students in general write to reproduce existing knowledge and to memorize facts (e.g., Danielsson, 2010; Osborne, 2002, 2007; Sørvik, 2015). Keeping in mind the transmissive tradition associated with school science, we find it crucial to ask students about why they are writing in the first place and to distinguish between, for example, texts that are used to make their own thoughts and ideas visible, texts that are used to help them obtain more information (e.g., e‐mails to a researcher who might have relevant knowledge about the project's main aim), and, finally, texts that are intended to present results to a broader audience. In the following, we will explain how we operationalize these three categories and we will provide examples from each of them.

*Thinking texts* are written mainly to clarify one's thoughts, both alone and within a group. The texts are directed inward and include, for example, logs, mind maps, and sketches. In this category, writing is done to “get ideas on paper” and functions as an aid to structure students’ own thinking and ideas.

**Figure 7:**
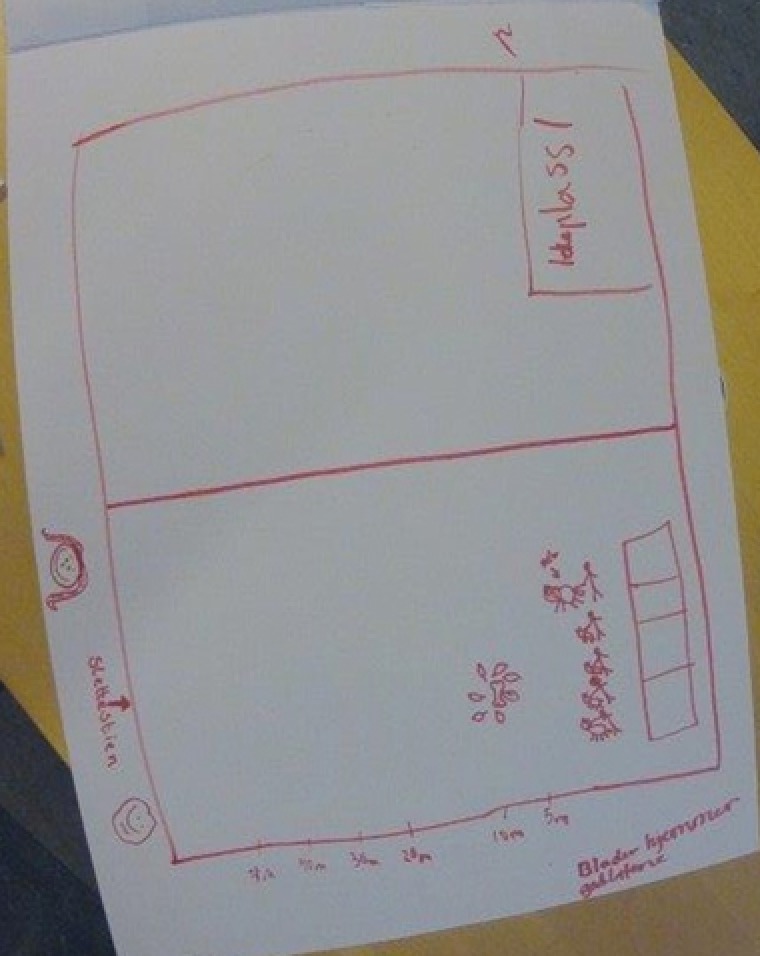
Example of thinking text, sketched map of dog experiment area [Color figure can be viewed at wileyonlinelibrary.com]

Figure 7 provides an example of a thinking text. The image of the text is from a head‐mounted camera, thus the bulgy lines. This text was made by a group of six students. It is a sketch of a map of the area where the students plan to conduct their experiments with dogs to test their hypothesis of the distance at which dogs can smell a treat. The words say “Slettestien,” which is the name of a local park, “Ideplass1,” probably meaning sports ground 1, and “Blader hjemmer godbitene,” which is a misspelling of “Leaves hiding the goodies.” After a short discussion on how they might conduct the dog experiments, the students started to draw with a broad red marker on a large flip‐over sheet. Four different students held the marker at some point, and all six students were active in the production of this multimodal text. This talking, writing, drawing, and thinking aloud resulted in a typical thinking text, meant for the authors to make their thoughts and plans visible.

*Working texts* are written mainly to obtain and handle information or artifacts. They are crucial to accomplishing important aspects of the research, typically in the data‐collection phase. This category includes, for instance, notes taken during experiments, e‐mails with questions for an expert, and a letter requesting the use of dogs for the scientific experiments. The texts are in a sense extrovert, directed at an audience, both inside and outside of the classroom, other than the student who wrote the text. Audience in the class includes, for instance, group members not writing the notes. Audience outside the class includes informants as experts on dogs, dog owners, and a pet shop employee. Notes taken during interviews, experiments, and reading are considered working texts because they served the function of supplying a group of students with relevant knowledge, written down by one representative of the group. Hence, the notes meet the notion of an extrovert text: the author did not write purely for him or herself, for instance, to clear his or her thoughts but to supply the group with relevant knowledge. Sometimes a group conducted an interview, with one student acting as secretary. On other occasions, the group split apart, read different sources, and shared notes afterwards. Both are examples of working texts: they are used to obtain information and supply the group with this knowledge.

**Figure 8:**
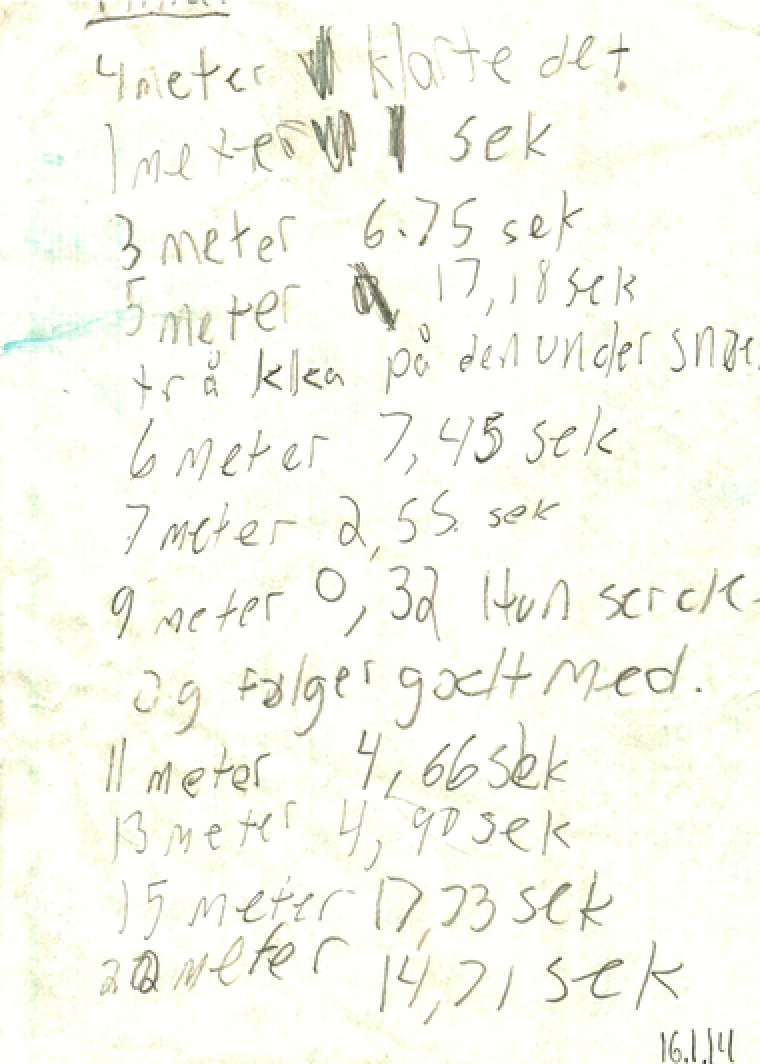
Example of working text, notes taken during dog experiment [Color figure can be viewed at wileyonlinelibrary.com]

Figure 8 gives an example of a working text. This is a handwritten note, written outdoors during an experiment with a dog. The experiment was conducted by a group of five students, one of which had the task of writing notes during the experiment. The text consists of results from the experiments, including the distance to where the treats were hidden, how much time the dog needed to find the treat, and some comments such as “Stepped on it under the snow.” This working text was used by the group to evaluate their experimental setup and by another group to compare the results of the first group with their own results. Hence, the text was characterized as a working text, extrovert and functioning to obtain information.

*Presentation texts* are primarily used to present a product and are also extrovert. In this category, we typically find articles, tables of results, and reports.

**Figure 9:**
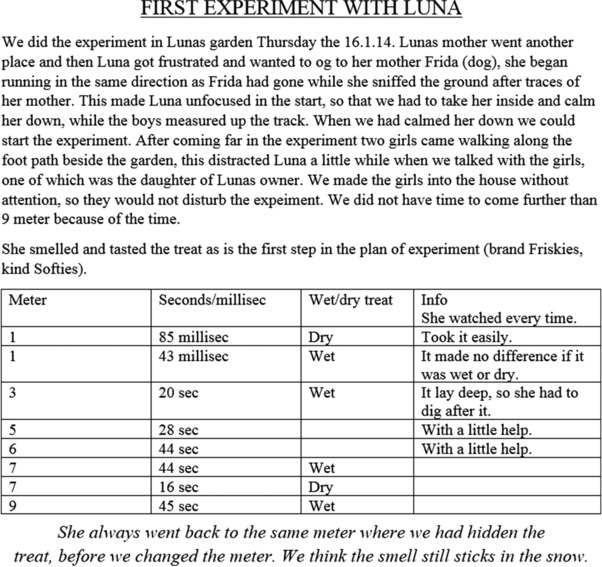
Example of presentation text, report on first experiment with Luna (a dog)

Figure 9 shows a text called “First experiment with Luna,” a report on a dog experiment. It is multimodal and contains prose, a table, and commentary in italics. This text was written as part of the main report, presenting the research to others, and contains a description of the context of this particular experiment, the results of the experiment, and a final comment.

These three categories, thinking, working, and presentation texts, are mutually exclusive and concern the main purpose of the texts for the author at the time of writing. All texts were categorized based on this initial or main purpose. Some of the texts were later reused in different settings by the students, especially in the report. For instance, some e‐mails, originally written as working texts, were later included in the report to document the data collection process. Although such texts, by way of appearance in the report, are made public, they are categorized as working text because of their original purpose. Consequently, not all texts in the report are presentation texts, and not all presentation texts are included in the final report.

#### 7.1.2 Why was the writing event initiated?

The second approach in our analysis concerns why the students started to write the different texts. The data‐driven categories, which are mutually exclusive, have writing events as the unit of analysis, including the text and the context. There are three categories:

*Writing instructions* were given explicitly by the teacher, for example, “Make an explanatory drawing of the dog's sense of smell.” The students were given a clear writing task but not always a specific‐intended genre.

*Open challenges* of different kinds were also given by the teacher, formulated through purpose, receivers, and/or context. For example, the teacher might challenge the students to find dogs to use for the experiments. The word “challenge” is used here because the objective was presented by the teacher to the class for such assignments. Importantly, a challenge does not necessarily include writing, because it was always up to the students to solve a given challenge in a manner they saw fit. The challenges included in the study material are the ones that resulted in writing.

*Student‐initiated texts* are written by the student without any prior encouragement or instruction from the teacher.

Categorizing the texts using the codes described above is not without challenges because some writing events may seem to fall between two categories. In such cases, the main criterion for sorting between “writing instruction” and “open challenge” was the teacher's wording. If the focus was mainly on a specific kind of text and the request from the teacher specifically mentioned writing, then the event was assigned to the category “writing instruction.” If the teacher described a purpose, problem, or situation the students needed to engage in during their research process, the event was assigned to the “open challenge” category. It should be repeated that the open challenges given by the teacher could be solved by actions other than writing, such as talking or reading, and the open challenges, therefore, do not clearly set the stage for writing in the same way as the previously mentioned writing instructions clearly do. When students write as a response to an open challenge, it is because they have actively chosen to do so.

Another issue is determining when a text was actually initiated. In some cases, there might have been a class discussion about a topic, and the next day, a student might have written something related to the topic. In other cases, the initiation of a text might not be possible to trace. As a rule, the initiation of a text is seen within the same day and it must be connected clearly to the text produced. Texts produced in the absence of clear initiative from the teacher were categorized as student initiatives.

In the analysis, texts were grouped and categorized based on the writing initiative. One instruction given by the teacher that resulted in 21 texts (one from each student) was counted as one writing event. Likewise, a student starting to write on their own was also counted as one writing event. This approach was chosen to emphasize the starting point of writing.

Additionally, we have analyzed the types of writing purposes that emerge from the different kinds of writing events in this study. The data from the two approaches were combined to obtain an overview of both the event and the intent of writing. This made it possible to look for patterns such as which writing purposes were most commonly associated with which writing events. It also made it possible to consider how teacher‐initiated writing events and student‐initiated writing events often serve different purposes.

It should be noted that our classification is qualitative, and we quantified text distribution to highlight tendencies and patterns in the material, not as an attempt to generalize these patterns.

## 8 RESULTS

The results are presented in accordance with the three research questions: First, we present our findings concerning the main purposes the texts served for the students. Second, we present our findings about what initiated the different writing events the students engaged in during the 8 weeks of the research process. We also combine these two sets of results to look at what kinds of text purposes emerge from the different writing events. Finally, we introduce a few findings based on the students’ own reflections on the entire research process as they relate to writing.

Table 4 presents a summarized overview of the 8‐week‐long research process and the texts written during that time. Not all steps or texts are included. The research roughly follows the inquiry process starting with finding research questions and posing hypotheses, then making plans for data collection, collecting data, and reflecting upon findings, and ending with concluding and communicating (National Research Council, 2012). Furthermore, the entire period is characterized by continuous writing of varied texts such as notes, lists, e‐mails, plans, summaries, reflections, articles, posters, and a conclusion. As much as two‐thirds of the total texts written were multimodal. In the final report, with longer and more complex texts, the multimodal texts amounted to three‐quarters of the texts. Details are presented in depth in the following sections.

**Table 4:** Overview of students’ research process and texts Written

Week	Research process	Texts written
1	Ideas for research question	Research questions
	Establish research question	Reflections about RQ
	Pose hypothesis	Hypothesis, summary of total hypothesis
	Try to get hold of dogs	Request, list of dogs
2	Prepare experiment on dogs	Plan for experiment, prepare sketch of experiment
	Prepare for contact with experts	List of questions and experts, mind map, e‐mails
	Test experiment on dog	Notes during experiment
	Regulated experiments on four dogs	Detailed plan for experiments, interview form to dog owners
3	Gather knowledge so far	Summary of class discussion, e‐mails
	Reflection about experiments	Reflections about experiments
	Interview experts	Interview guide, notes
	Second round of dog experiments	Recipe for dog experiments, notes
4	Discussions about experiments	Notes from plenary discussion
	Reflections about hypothesis, was it lacking	Notes, tables
	Third round of dog experiments	Notes, form for notes
5	Collecting facts about the smelling sense of dogs	Notes
	Make “Did‐you‐know” posters	Posters
	Reflections about the research so far	Individual logs
6	Class away on camp	
7	Gather all information from experiments	Summary
	E‐mails from interviewed experts examined	Highlighting text, keywords
	Work on article on dog's olfaction	Article
	Conclude texts on experiments	Summary
8	Discussion about hypothesis and findings	Draft and final conclusion
	Design report	Front page, table of contents, preface
	Revision and submission of report	Proofreading/editing, revision

### 8.1 What purposes do the students’ texts serve?

**Figure 10:**
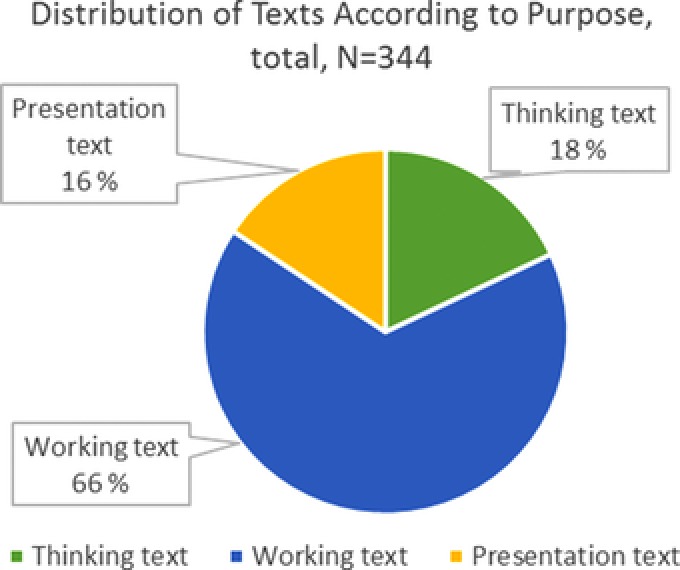
Distribution of texts according to purpose [Color figure can be viewed at wileyonlinelibrary.com]

In total, 344 texts were written by the students during the study period. As shown in Figure 10, the majority of the texts, 66%, are *working texts*. These texts were prepared as part of the research process, mainly to gather data. Such a distribution suggests that the students had time to battle with ideas, handle “messy data,” and improve their hypothesis, methods, and texts. The thinking texts and presentation texts are distributed somewhat equally.

**Figure 11:**
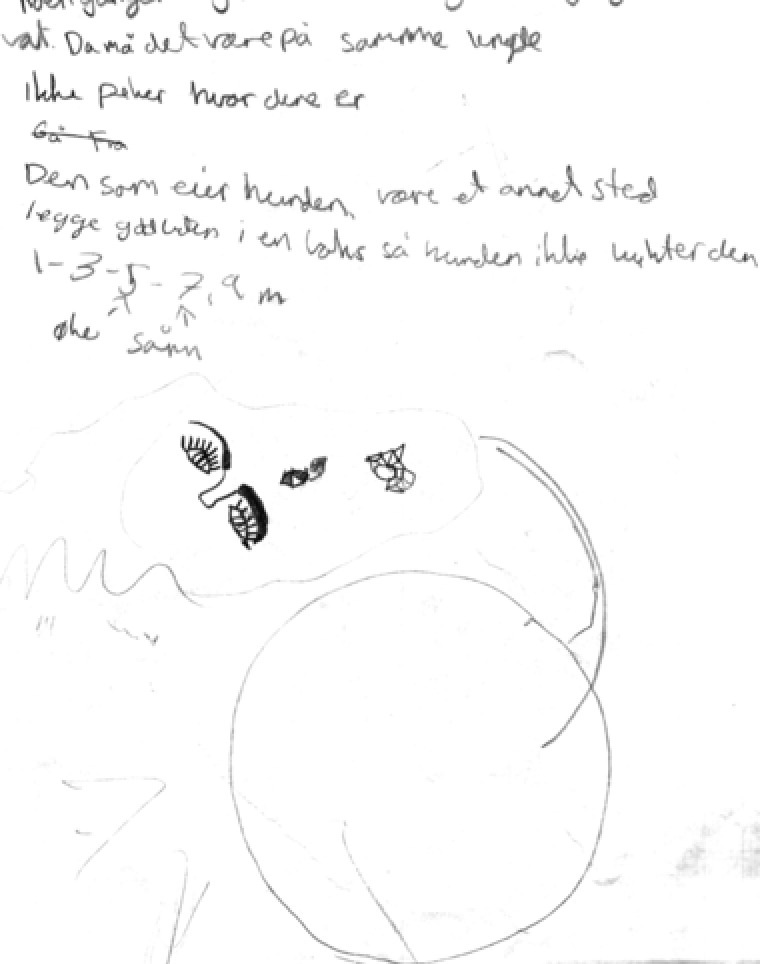
Example of thinking text, for dog experiment. “It must be the same distance. Don't point to where it is. Go forewa The dog's owner should be somewhere else. Place the treat in a box to avoid the dog smelling it. 1‐3‐5‐7, 9‐ increases this”

**Figure 12:**
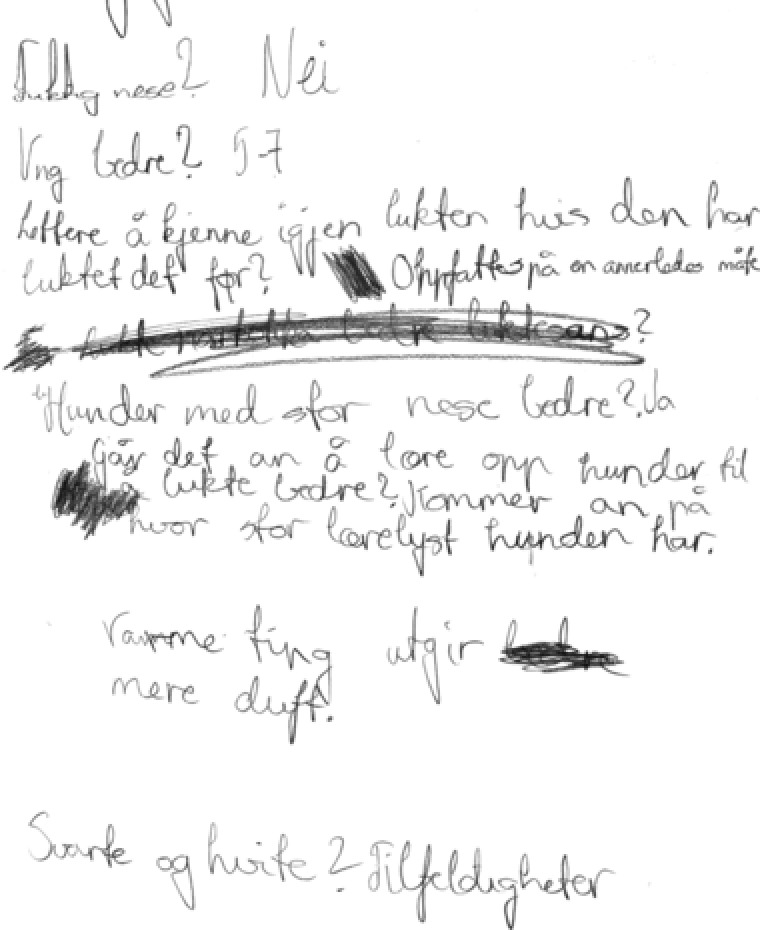
Example of thinking text, reflections on data, and hypothesis

*Thinking texts*, making up 18% of the total texts, are written at different stages in the research process. We find, for instance, thoughts about how a possible research question could work out in the initial phase. Sketches and ideas of how to conduct the dog experiments show up as the students work with research design (Figure 11). During the data collection period, some students reflected on the findings up to that point in terms of their connections to the different hypotheses (Figure 12). Finally, we find thinking texts connected to planning of the report's front page.

**Figure 13:**
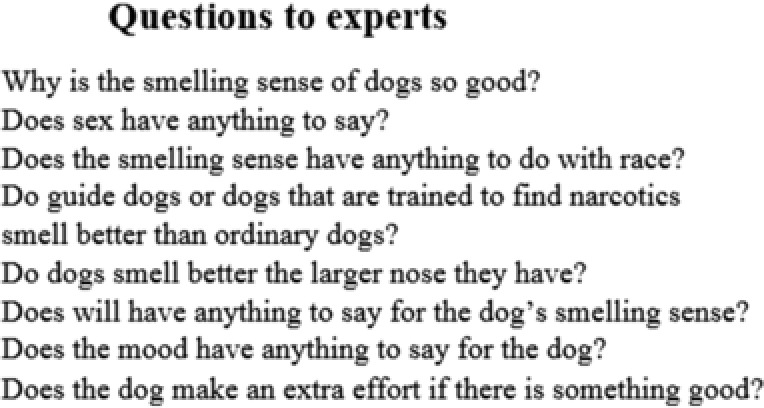
Example of working text, interview guide “Questions to experts”

**Figure 14:**
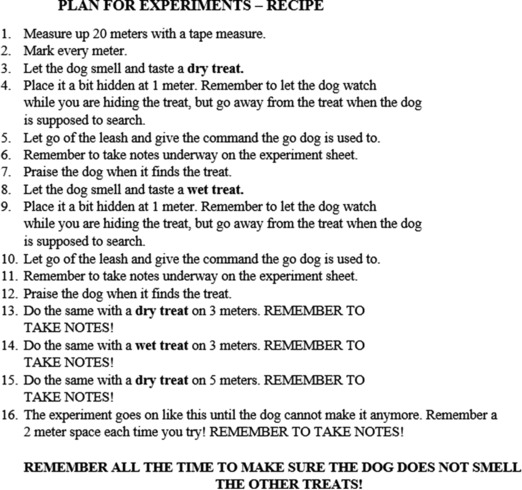
Example of working text, “Plan for experiments – recipe.”

The 66% of texts classified as *working texts* played a part mainly during the periods of research design and data gathering. Many of the texts are notes, taken during experiments, telephone, or face‐to‐face interviews or after a discussion in class about relevant facts. Some notes are more structured, such as forms for interviewing dog owners and tables for recording data for the second round of dog experiments. E‐mails to experts are included in this category, as is the interview guide used for all expert inquiries (Figure 13). A plan for experimentation with detailed descriptions of how to conduct the experiments was also written (Figure 14). Other working texts include the manuscript of a documentary film on the students’ own research, the actual film, and drawings intended for film cover. Taken together, these texts cover diverse writing practices that are known to researchers as being necessary to conduct a research project.

**Figure 15:**
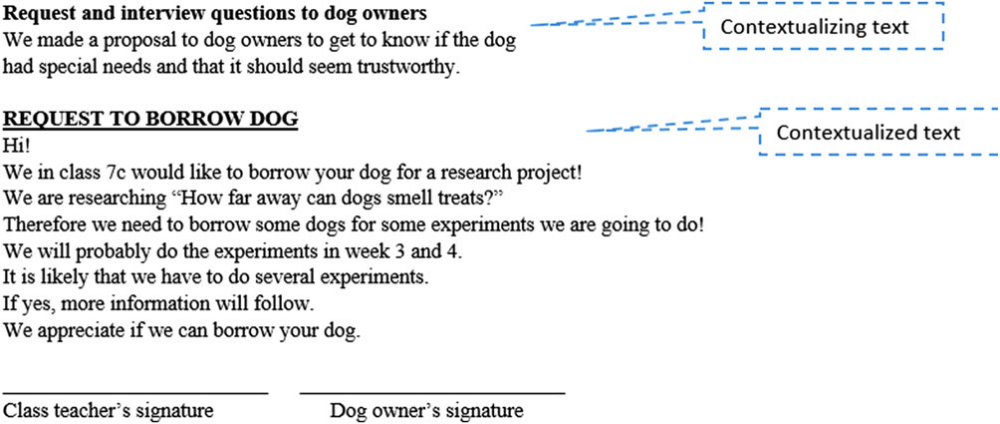
Example of working text included in the report, “Request and interview questions to dog owners” [Color figure can be viewed at wileyonlinelibrary.com]

The working texts can be further divided into two groups depending on whether they are included in the final report. Less than half of the working texts were included in the final report. An example of included texts is the request to dog owners to borrow dogs (Figure 15). The request was distributed to neighbors who had dogs. Later, the written request, accompanied by contextualizing text, was included in the final report. The contextualization in this example is implicit, functioning as an introduction to the text. There are several examples of working texts clearly glued into the report, often with different font size and layout than the introductory words. When these texts are included in the report, it is precisely to underscore their role as working texts and to show the broader audience how the students conducted their research. This aligns well with the ideals of methodological transparency in science and research in general.

Some e‐mails and an interview guide were attached at the end of the report. This matches the praxis of other scientific reports, where some texts essential to the research are appended, to elaborate, for instance, details of the method used.

**Figure 16:**
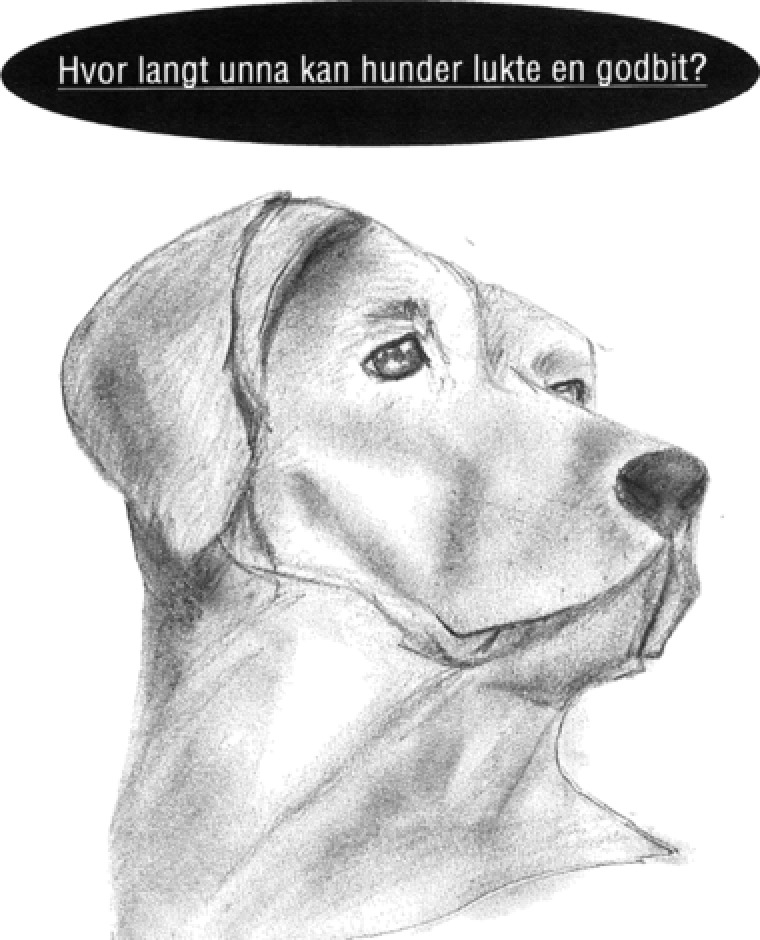
Example of presentation text, front page of report, “How far away can dogs smell a treat?”

**Figure 17:**
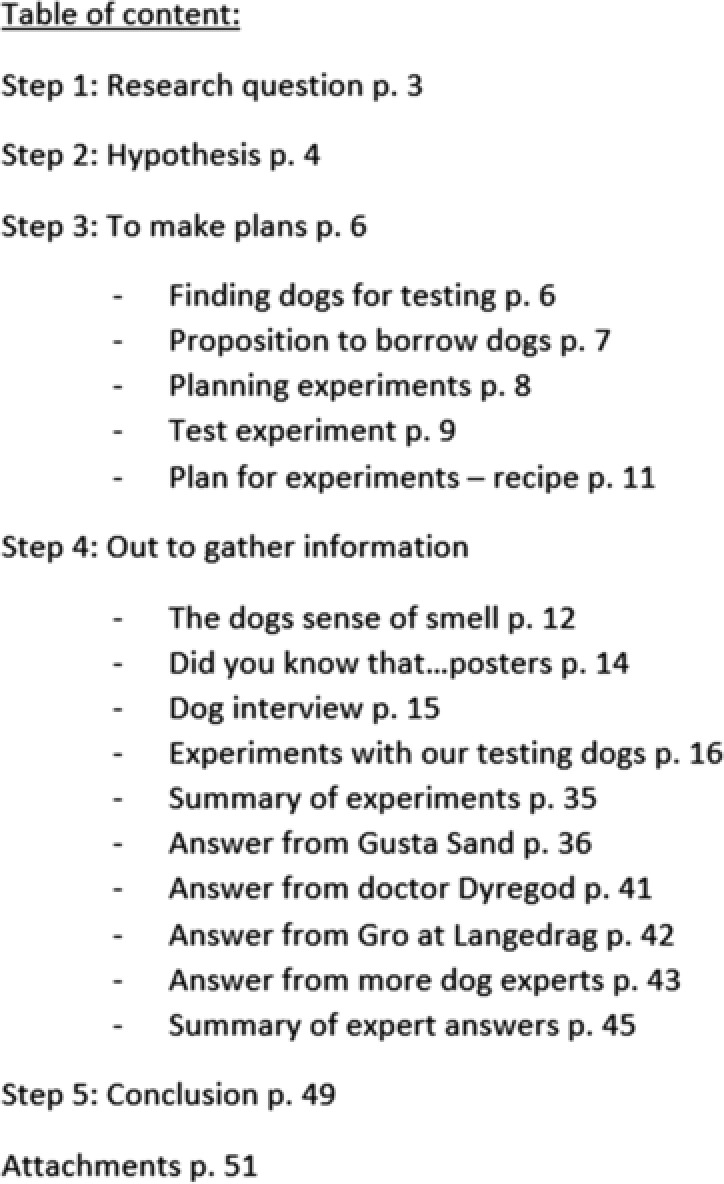
Example of presentation text, “Table of contents”

*Presentation texts*, making up 16% of the texts, consist mainly of texts written specifically for the final report. The report submitted to the research contest consisted of 54 typed pages. A front page with a pencil drawing of a dog (Figure 16), a preface, and a table of contents frames the rest of the report, which is organized according to the five steps advocated by the contest for describing the research method. The table of contents, as written in the report, is shown in Figure 17.

**Figure 18:**
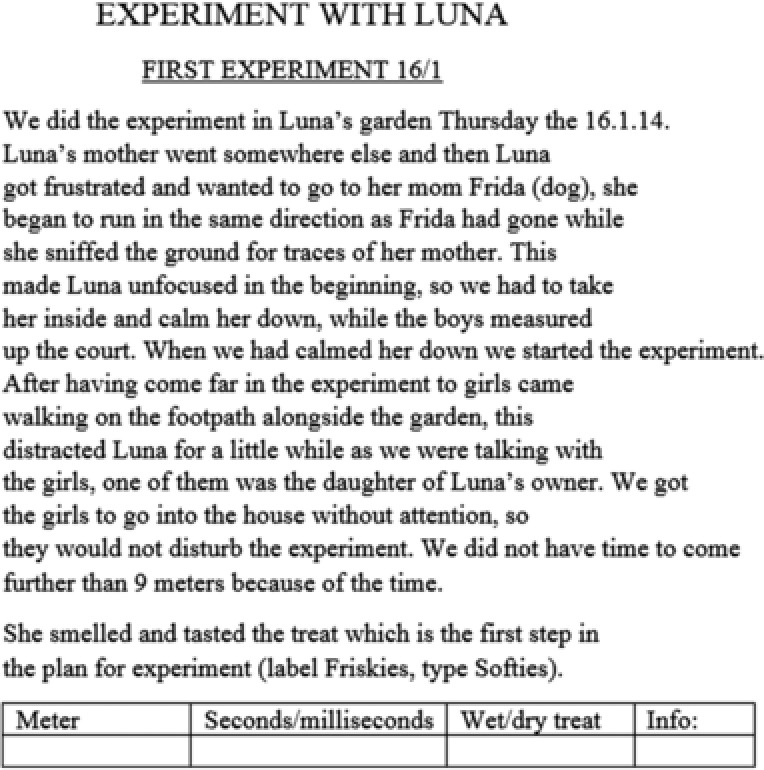
Example of presentation text, “Experiment with Luna”

**Figure 19:**
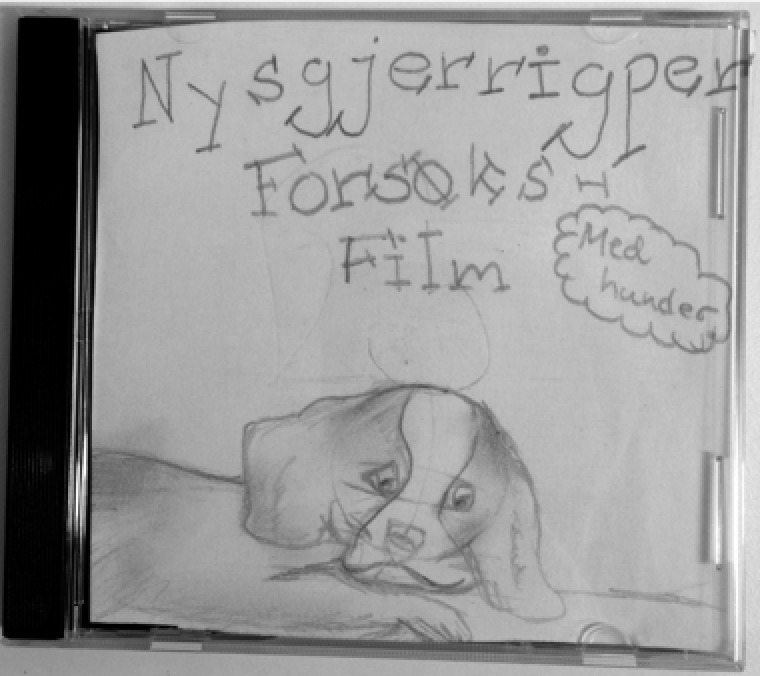
Example of presentation text, cover, “Research movie with dogs”

Organization of the report corresponds with the basic approach of the hypothetico‐deductive method. A research question is posed, accompanied by a hypothesis. The plan for data collection (Step 3) covers planning experiments with dogs, including gaining access to dogs that could participate in the experiment. Step 4 involves gathering information, both from the experiments and from different experts, on the olfaction of dogs. The report ends with a conclusion and attachments, which include e‐mails to experts and the interview guide. Presentation texts in the report include the front page, the table of contents, the preface, reports on dog experiments (Figure 18), and the conclusions. A cover for the documentary film also serves as a presentation text (Figure 19).

### 8.2 Why do the students write? Initiative of writing

#### 8.2.1 Distribution of initiative of writing events

The distribution of writing events is sorted based on the initiative for writing, that is, the initial phase of writing is analyzed to determine whether it comes from the teacher addressing one or several students or from the students themselves. In total, there are 80 writing events, including writing instructions, open challenges, and student initiatives, as described in the Data Analysis section. Most of the instructions and challenges were given orally by the teacher during plenary situations. Three lessons of the 26 total lessons were dominated by written instructions or challenges. In some cases, the teacher gave instructions or challenges to single students or groups of students.

**Figure 20:**
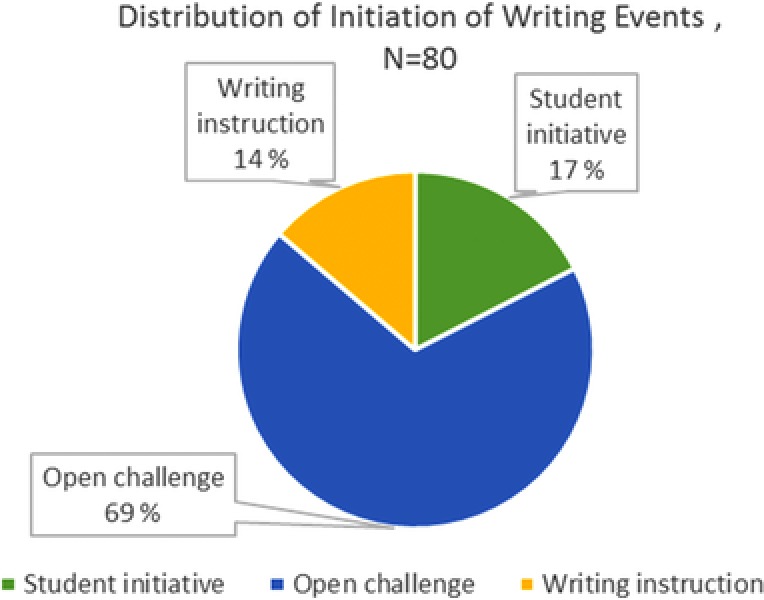
Distribution of writing events according to initiative [Color figure can be viewed at wileyonlinelibrary.com]

From Figure 20, we can see that the tasks given as open challenges, constituting 69% of the writing events, dominate the initiation of writing. In these instances, the students themselves decided to address the challenge through writing. Thus, a clear majority of the texts were started because the students themselves chose to write, not because the teacher provided them with a direct writing task. Only a minority of the writing events, 14%, were a direct result of the teacher's instruction, a finding that underscores the role of the teacher as a facilitator and supervisor.

#### 8.2.2 Writing instruction

The small proportion of 14% of tasks initiated as writing instruction, where the teacher clearly tells the students to write, was typically placed in context and often directed at the entire class, as Example 1 illustrates. The event occurred early in the research process when the class had just agreed that they would investigate the research question “How far away can dogs smell treats?” The teacher guided the class toward the next step in the research process, namely, the creation of a hypothesis.

*Example 1, Writing instruction*

Teacher: Then, you are going to write down your hypothesis on the green sheet of paper given to the group … It is really important that you be open to different answers because most questions have several answers.

This task ended with about 25 different hypotheses, which were then reduced to 13, because some had the same content with different wordings; the hypotheses were then summarized on one sheet.

As a strategy to remember or document their work, the teacher asked various students to function as “secretary” during plenary discussions, especially when such discussions were centered on gathering information.

*Example 2, Writing instruction*

Teacher (to a specific student): Would you mind being secretary when we discuss the dog's sense of smell in class? Writing down what the class says?

The students usually responded positively to this type of request to write. Some chose to take notes by hand, others on the computer. Some students wrote whole sentences, others wrote notes using keywords, and some included arrows, signs, and quick drawings.

#### 8.2.3 Open challenge

The majority of the tasks (69%) were challenges given by the teacher. As explained in the analytical framework, these challenges do not require writing per se, but students can choose to use writing to solve the challenge. In Example 3, one day, in a plenary session, several groups presented their findings, answers from various experts such as veterinarians, pet shop employees, and a police officer from the narcotics department. The next day, the teacher presented written challenges on the interactive board in the classroom and asked the students to sign up for the various challenges.

*Example 3, Open challenge*

Challenge: Can you organize the answers of the experts?

The challenge was in this case given as a question. The purpose was to organize the answers of the experts, with no clear directions for accomplishing the task. Two students took this challenge and started to write on the computer the answers given by various experts and started a discussion.

Joachim: We can write like this: Why, blah blah blah. And then Eirik answered: … And then we write that.
Adam: Yes, the answer and things like that.

The text written for this open challenge ended up with the heading “Answers from dog experts” and a short introduction stating that several experts had been asked questions and had given various answers. Different questions, marked with boldface and underline, were presented, including answers from three different experts. The text was clearly organized, and the answers of the different experts were easily comparable.

After the class decided on which of the hypotheses to investigate, the teacher challenged the students to come up with ideas on how the dog experiments could be conducted. Some features of the content were mentioned, such as the hypothesis as starting point, measurements, and the research question. The purpose here was to think—nothing was to be decided.

*Example 4, Open challenge*

Could someone think through how the experiments could be done? The hypothesis must be the starting point. We would probably need to measure some lengths here because we are investigating how far away dogs can smell objects.

The open challenge is formulated to get students to think about possibilities. A rather large group of eight students joined in on this task. They ended up drawing plans indicating where and how to perform three different versions of the experiments. They also wrote a five‐point plan on how to conduct the experiments.

In a later phase, several challenges, written by the teacher, were given to the class, most of them formulated as questions. Example 5 shows a written challenge: explain how the experiments are done. This challenge was the outcome of a long discussion of some notes taken during the initial experiments, which were so different that the students understood they could not draw any conclusions or even compare the findings. To describe exactly how the experiments were to be performed was therefore important for the upcoming experiments and the trustworthiness of the research.

*Example 5, Open challenge*

Challenge: How are the experiments done?

Two different groups took this challenge. One group used a camera, took pictures of the intended way to perform the experiment, and combined the pictures with a movie another student was making for their research. The other group wrote a detailed plan called “Recipe” (Figure 14) for conducting the experiments and included a sketch of the experimental setup (Figure 11). They also presented to the class a step‐by‐step demonstration of how to conduct the experiment. The same open challenge was therefore solved differently, but adequately, by two different groups, showing that the students’ choices are significant in the process and in their choice of writing practices/events.

#### 8.2.4 Student initiative

Writing events categorized as student initiatives, comprising 17% of the events, include texts where the teacher clearly played no part in initiating the work and the student made the decision individually to start writing. Most cases of student initiatives are notes to plan, gather, or sort data. We found notes from experiments; from interviews, both face to face and by phone; and from reading books, articles, webpages, and the students’ own texts.

In Example 6, the teacher approaches two girls who have written a request to dog owners to borrow their dogs (Figure 15). The teacher is concerned with this text, which was the result of a challenge to obtain dogs to participate in the experiments, but one of the girls has another initiative as well.

*Example 6, Student initiative*

Teacher: (Reads the request to dog owners.) Yes. Really good.
Randi: And now I have to make a sheet with questions. About the dogs.
Teacher: Yes, that is probably a good idea. But this is the most important part.

The teacher is pleased with the students’ choice to write a request as a solution to the challenge of getting hold of dogs and is focused on that. However, Randi (the student) is already planning her next step. She wanted to write a sheet with questions for the dog owners. This was her initiative; the teacher acknowledged it but remained focused on the original request. The teacher moved away from their desks after this, and Randi started on the questions she had thought of. At the end of the lesson, when all the groups gathered to present their work for the class, Randi and her fellow writers presented both the request and a list of potential questions for dog owners. The next day, another student, Olav, joined the group to complete the text, after which this interview was conducted:

Interviewer: Can you explain what you've got on that sheet of paper?
Randi: It's for an interview with the dog owner.
Vilma: They're questions. We're going to ask the dog owner questions.
Olav: About the dog, that is.
Interviewer: Why are you doing that?
Randi: We have to know a little about the dog. And if it has any diseases or something, we should take that into consideration in the research, when we are doing experiments. And they [the questions] are based on the hypotheses, we have questions about them.
Interviewer: Why did you find exactly these questions?
Vilma: We got the questions out of our hypotheses. For instance, the age… We believe that when the dog gets older, their senses get weaker. So therefore, we ask about the age of the dog.
Randi: And then, for instance: “Is the dog actively hunting?” Because one hypothesis is that hunting dogs have a better sense of smell.
Vilma: Well, not better, but it is trained to search and stuff. It's not just better. I think they are born just the same. They are not born as a hunting dog, they turn into one and are taught [how to hunt]. And then maybe their sense of smell gets better.
Randi: “Is your dog trained to search for things?”
Interviewer: You called this an interview. How have you planned to do it?
Randi: We are going to do the interview and write down [the answers]. We need it for our research.

The previous list of questions has now resulted in some potential questions for an interview with dog owners. The students explained clearly that the questions are there to test the hypotheses and support their research. As made evident by Vilma's elaboration about why they want to know the age of the dogs they use in their research, a simple question about the dog's age is rooted in their project, designed to obtain information relevant to testing their hypotheses. Another question, about the connection between hunting dogs and sense of smell, was also discussed. Further, the modalities chosen were not coincidental. As Randi explained at the end, the interview is to be done orally, with the written questions as a starting point, and then the answers provided by the dog owners will be written down. This showed an awareness of different representations of information and that the students deliberately chose writing and orality.

### 8.3 What kind of text purposes emerge from the different writing events?

**Figure 21:**
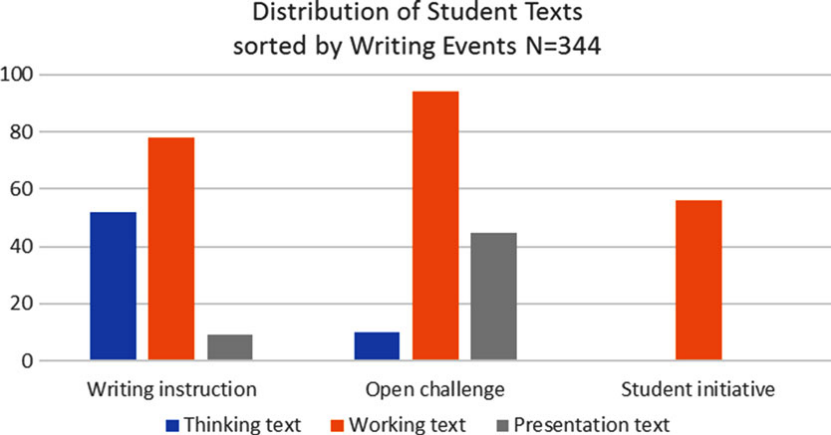
Distribution of student texts sorted by writing event [Color figure can be viewed at wileyonlinelibrary.com]

The data presented in Table 5 and Figure 21 show how many texts within the different categories of thinking texts, working texts, and presentation texts are the result of the three types of writing events: writing instruction, open challenge, and student initiative.

**Table 5:** Distribution of student texts sorted by writing events

Category	Thinking text	Working text	Presentation text	Sum
Writing instruction	52	78	9	139
Open challenge	10	94	45	149
Student initiative	0	56	0	56
Sum	62	228	54	344

When we analyze how many of the writing events are a result of the teacher's writing instructions, we find that this is the case for only 14% of the writing events. Still, 139 of the total 344 texts (40%) are the result of writing instruction given by the teacher. This makes sense when we take into account that the writing events where the teacher prompts the students to write happen in whole‐class settings where all students subsequently produce texts associated with the task provided by the teacher. Of the 139 texts associated with writing instruction by the teacher, 52 are thinking texts, all of which were produced at the beginning of the project either to find a suitable research question or to establish workable hypotheses.

**Figure 22:**
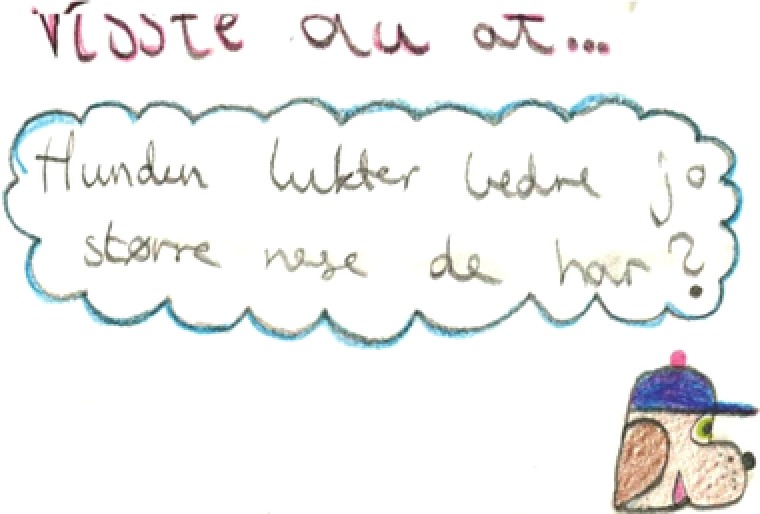
Example of teacher‐initiated text, Did‐you‐know‐poster, “Did you know that… among dogs the bigger the nose, the better the sense of smell?” [Color figure can be viewed at wileyonlinelibrary.com]

Among the 78 working texts, as many as 47 were posters made to inspire curiosity about the class’ research on dogs among other students at the school and to develop a mutual understanding of the important findings so far in the project. All the posters were multimodal and consisted of the heading “Did you know …?,” followed by a fun fact and a drawing. An example is included in Figure 22.

The presentation texts, nine in total resulting directly from teacher instructions to the students to write, were, not surprisingly, written at the end of the project. Of these, eight were summaries of findings strengthening or weakening the hypotheses and were all written at the same time. This was a demanding task for the students, and it required more guidance from the teacher than most of the other writing events observed.

To sum up, the writing instructions, where the teacher explicitly asked the students to write, although they amounted for only 14% of the writing events, were central in the research process. They were crucial to initiating a sustainable research process, developing a mutual understanding of the project during its course, and seeing the bigger picture in the complex and messy data and its relation to the many hypotheses the students originally generated. Hence, the teacher's writing instructions framed the research process and resulted in thinking texts, working texts, and presentation texts.

**Figure 23:**
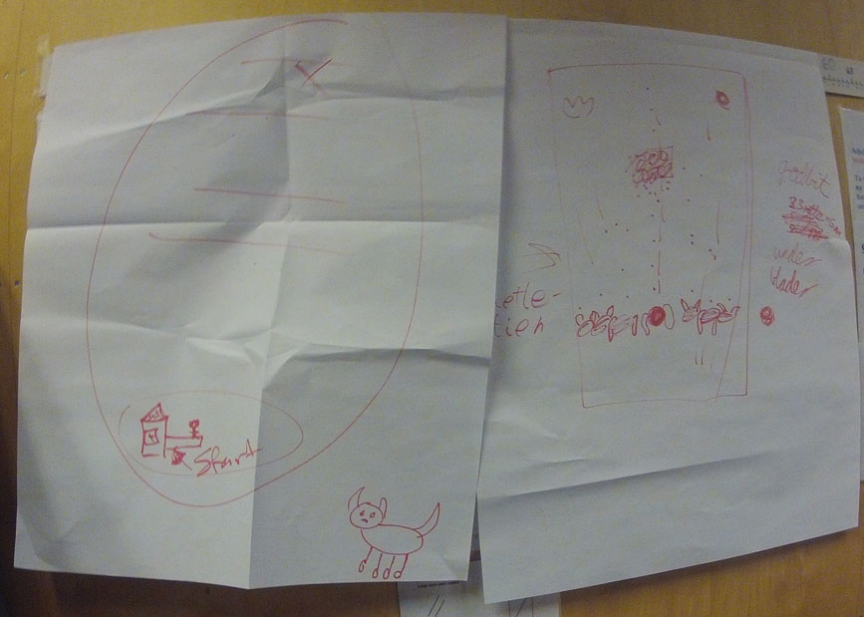
Example of text from open challenge, sketch of dog experiment [Color figure can be viewed at wileyonlinelibrary.com]

Written responses to open challenges constituted the biggest and most varied group of texts, encompassing 149 of the 344 texts in total. Of these, ten were thinking texts, nine of which included different sketches and loose plans on how to conduct different kinds of experiments to test the sense of smell of dogs. Several of these texts were multimodal, as they included both drawings, arrows, words, and sentences (see Figure 23). By far, the largest type of text generated from the open challenges was working texts (94). No special writing event dominates; rather, there seem to be working texts throughout the project, especially to plan for, sort, and understand the data. Some examples are texts to organize the finding and borrowing of dogs and the information about them. Questions to experts and organizations of the answers are also frequent in this group. From the total of 54 presentation texts, as many as 45 were generated as a result of an open challenge. This category consists of texts presenting the research, following a chronological order, including how the research question was found, the methods used to gather data, the results, and the conclusion. Reports about the experiments on the different dogs account for 14 of these texts. To sum up, the open challenges are given throughout the research period. Thinking texts were primarily used to plan experiments. The working texts functioned primarily to gather and understand data. The established types of text included in scientific reports were typical presentational texts that arose from open challenges.

The student‐initiated texts, 56 in total, were all categorized as working texts. These texts were mainly used to plan data collection, record notes during interviews, experiments, and reading, and finally, use notes to get an understanding of the results. We find it noteworthy that no thinking text was initiated by the students. Further, no presentation texts were generated as a result of a student initiative.

### 8.4 Choosing to write

The aforementioned request written to dog owners (Figure 15) was the result of an open challenge to find dogs to use in the experiments. The two students who had taken the challenge were interviewed about their choice of using writing to obtain dogs for the experiment. After talking with the students about a list of potential dogs, the interviewer (the first author) asked specifically about the written request:

Interviewer: Good. Let's have a look at the other text. What is it?
Vilma: It's the request we're going to give to the owners of dog we'll ask to borrow. Well, a request to borrow dogs.
Interviewer: But why did you want to make a written request?
Randi: Because it's like … it could be that we were only kidding, for instance. So, they'll get a better impression, well, when we make this kind of request than if we were only going to say it like: “Can we borrow your dog?” I think… And when we write that [points in the text], they know it's not just for fun.
Interviewer: What did you point at there?
Randi: The signature of the class teacher.
Vilma: It is important that they understand it's real and not just kidding, wanting to take the dog.
Interviewer: It seems smart, to show that you're serious.
Vilma: It would be stupid if they thought we are fooling around, and so they may not lend us their dog because of that.
Randi: And they'll get a better impression of us as well, and it is a bit important that we show that we won't fool around with the dog, but do it properly.

As we can see, the student Vilma introduced the word “request” and explained the purpose of the text, to convince dog owners to lend their dog to the students. Randi stated that the owners would have a better impression if they were approached with a written request, not just an oral appeal. The students were aware of different options to solve the challenge, but they chose to write. Thus, the selection of the written mode was conscious and reasoned. A written request signaled something “real,” as opposed to “just kidding.”

### 8.5 Writing practices among students as researchers

Toward the end of the research project, four groups of students were interviewed and were asked for their experience of science lessons in which they could work as researchers. This was done because reflections on and conceptions of writing can shed light on the underlying writing practices in the class. The excerpts presented below are representative of these interviews because they revolve around the usefulness of being a student researcher. A group of two students interviewed about a report on a dog experiment were asked “Now you have worked as researchers for a while. What do you think you've learned?” Here is an excerpt from the students’ answers that directly concerns writing.

Randi: And we learned how to write a report and so on. When you are doing research, it leads to a lot of writing. So, we learned to organize, write requests, because we will surely need to do so when working by ourselves.
Interviewer: What do you mean?
Randi: I mean when we get older and get a job on our own. Because in a lot of jobs, you have to write a lot.
Interviewer: So, what are you thinking? Is it more like a job?
Randi: Yes, we will need it later. So, we have written reports.
Ulrik: People who work write e‐mails all the time. I suppose you do, don't you?

When asked to say something about acting as a student researcher, Randi responded that conducting research involves a lot of writing, and she links writing to different parts of the research process. Moreover, she also had the expectation that the writing practices and organizing learned in the process would be useful when the students start work as adults. Both she and Ulrik agreed that adults write a lot in their jobs, and by acting as researchers, the students were gaining experience with writing that could be useful later in their adult careers.

Another group presented similar thoughts on the same question, namely “Now you have worked as researchers for a while. What do you think you learned by working like this?”

Karen: A little about how to manage on our own after school. When you are done with your education and live by yourself, alone.
Interviewer: What do you mean?
Karen: Maybe a little about how to do things and take initiative. It's like that at work, when you get a bit older. Then it's not like this: do this, these tasks. Then it's a little more like, write this text, do it like you want to. Make your own presentation.
Adam: Yes, that you can make it yourselves and don't have to follow things. That you can write what you want, like the presentation, as long as it is within what you are writing about.

In this group, Karen also emphasized the connection between their work at school and their research process, as well as its relevance for later work. She expressed that the way in which the task was given in their ongoing research work probably resembled the way it is done in the adult world. “Then it's not like this: do this, these tasks. Then it's a little more like, write this text, do it like you want to. Make your own presentation.” Adam supported this view; he said that you can make, for instance, a presentation yourself, the way you want. To a follow‐up question on what “things” you “don't have to follow,” he explained writing frames that the teacher would normally give them. These students’ conception of “work” is associated with white‐collar jobs, where higher education is expected and individuals are given responsibility.

As all the interviews from the entire period revolved around the initiation of writing, the students answered the question of why they wrote. Typical answers were to remember, to get more information, or to present information. On a few occasions, when a writing event was categorized as *writing instruction*, the students pointed to the teacher as their reason for writing. Not surprisingly, we identified that the students’ reflections on the purpose of writing are clear and in line with purposes outside the school context when a text is the result of an open challenge or student initiative, but the purpose and connection with a larger context outside of school is not always as readily evident when a text is the result of a writing instruction.

## 9 DISCUSSION

In this study, we aimed to identify the writing events in which students in elementary school engage when conducting research. More specifically, we wanted to investigate what and why students as researchers write when given an opportunity to actively engage in exploring their own scientifically oriented questions. We have not analyzed the texts per se, with regard to modality, linguistics, or content, or developments in their disciplinary writing, but rather conditions that motivate students to partake in disciplinary writing.

A key finding of the present study is that students write many texts, in all phases of their research process, covering a wide range of writing genres, and for different purposes. Most of the written texts were not used in the final research report on which the students were working, even though the written report was the contribution to the contest in which they were participating. This finding suggests that these students engaged in a range of writing practices similar to those typical of scientists (Howes et al., 2009; Yore et al., 2004). Although the goal of a scientist often is to publish, be it a report or a journal article, a lot of writing is undertaken to plan processes, obtain information, document experiments, and experiment with different versions of data, presentation, and so on, in addition to revising the texts intended for publishing. Literacy, in this case, writing, is a central aspect of being a scientist, and our findings suggest that the participating students engaged not only in traditional “schooled literacy” (Sørvik et al., 2015) but also in writing practices labeled as more authentic by the students. We found that many of the students’ texts serve functions that are not typical of science writing in school. Most of the students’ texts were not written for evaluation or to reproduce facts (Danielsson, 2010; Osborne, 2007), as is typical in school literacy, but as part of an ongoing process to capture thoughts, build ideas, and gather information that is more complex than simply transmitting information. Hence, the findings in this study illustrate the potential for the generation of a variety of texts and continuous writing through inquiry learning, approaching the description of scientific practices given by National Research Council (2012, p. 27) as defining data, analyzing, and revising as a fundamental social enterprise, both during the research and when communicating it.

Narrowing down to text level, the students in this study favored writing, more often than not multimodal, to handle large amounts of information in planning, gathering, understanding, and presenting complex data. Three steps to scientific writing can be inferred: The first step, internalized writing practices, was student initiated and revolved around handling information through note taking. The second step, potential writing practices, was triggered by open challenges and included writing that was recognizable to students from earlier experiences as researching students, such as text types in scientific reports, sketches for experiments, and structuring tables. The third step, scaffolded writing practices, was based on instruction by the teacher and consisted of whole‐class writing giving guidance at crucial stages of the ongoing research. This ladder step, with increasing text complexity and the need for scaffolding, underscores that socialization into scientific writing takes years. Still, the students in this study, performing mainly on the first and second steps, show that a high level of student autonomy can be achieved at relatively early ages if they are supported with well‐staged guidance.

In the following discussion, we highlight some issues we believe are of particular importance for successful integration of writing and science instruction. First, we discuss the benefits of student‐initiated writing. Then, we discuss the students’ writing as a way to approach the work of scientists. Furthermore, the overarching context, students as researchers, is discussed, both from the students’ perspective and in terms of educative value and limitations.

Given that a great deal of writing was undertaken, an important finding in the present study is that the writing of most of the texts was initiated by the students because they either *chose* to write as a result of an open challenge (when given the opportunity to solve tasks without writing) or as a student initiative. Thus, the writing was out of the control of the teacher, who could not plan to assign specific types of texts or features within scientific writing, such as argumentation. We acknowledge, however, that the teacher played a crucial role in providing opportunities for inquiry and challenges that guided the students through the research process and gave them opportunities to use writing in meaningful ways. This finding is opposed to other research indicating that instruction is the dominant way of initiating writing in school in general and in science in particular (Lyons, 2006; Sørvik & Mork, 2015). Further, our findings are in stark contrast to other studies in which science and literacy were deliberately integrated and planned for (Cervetti et al., 2012; Ødegaard et al., 2014; Peck, 2010; Sampson et al., 2013). In these latter studies, the researchers and/or teachers carefully planned how to write (for instance, argumentative), the concepts on which to focus, or the kind of text to write. Although the students were seldom directly told to write in our study, one might argue that they were expected to write, as the final product was supposed to be a written report. This might be the case for the final report and the presentation texts, but we found far more writing, and for further purposes, than one would expect for the final report. In some occasions, the students themselves pointed to their active choice of writing and showed awareness of other alternatives, such as orality. Further, other options than writing were also chosen, such as video and oral presentations or discussions, but as these other modes that did not involve writing were not our focus, these events are not highlighted in this article. Finally, we are aware that the scientific culture is heavily dependent on text (Lemke, 1998; Norris & Phillips, 2003; Osborne, 2007), a notion that may be passed on to students tacitly throughout elementary school. To state that writing was chosen totally freely would therefore be an exaggeration, once the context is taken into account.

The students in our study met what Sampson et al. (2013), p. 666) refer to as more authentic writing in terms of being realistic and embedded in the inquiry process, but not as educative, as no instructor was modeling or scaffolding the students’ work. This implies that given the right circumstances, students can take initiative to write (even without instruction or scaffolding) because they consider writing a viable part of their research process, as emphasized by Garcia‐Mila et al. (2011). Authentic writing has been found to relate strongly to growth in students’ ability to read and write texts of various scientific genres (Purcell‐Gates et al., 2007).

One can debate whether it is possible to conduct authentic writing within school, given the educative purpose of school (Barton, 2007, p. 127); such debate reflects an understanding that writing in school is different from writing in other arenas. Yet, if the notion of authenticity is viewed in the light of every writing event and not restricted to school literacy per se, there might be instances of authentic writing in school. We view authentic writing in line with Purcell‐Gates’ et al. (2007 p. 14) definition, which states (a) that texts are written for the same purpose as an outside learning‐to‐read‐and‐write context and (b) that a writing event serves a socially meaningful purpose. In the present study, the students wrote a request to ask for something and made notes to remember things. We argue that they thereby were engaged in authentic literacy practices, which have proven to be beneficial to enhance genre competence in elementary school classrooms (Purcell‐Gates et al., 2007). Hence, the situation caused the students to participate in more authentic writing practices within the school context.

Writing, in the present study, revolved mainly around clearing thoughts, gathering data, and presenting research findings in a written report. Such thinking texts play an important role when thoughts and ideas are complex or incomplete. It is a challenging but important stage in approximating the work of scientists, who often deal with unclear data (Osborne, 2007). These purposes can be seen as an epistemologic aspect of writing, where writing functions as a process that leads to construction of understanding (Hand, Gunel, & Ulu, 2009). Furthermore, the acts of gathering data and presenting a study report reflect the fundamental sense of scientific literacy (Norris & Phillips, 2003). By working with concepts and reasoning within the discipline, it also reflects the epistemic aspects of writing (Klein & Boscolo, 2016). Thus, the notion of writing to learn, especially by socializing into the world of scientific inquiry, is approached (Klein & Boscolo, 2016). The students’ writing thereby becomes a way of experiencing scientists’ work and participating in the text practices of science (Barton, 2007; Sørvik & Mork, 2015). The large proportion of multimodal texts in the study resembles scientific writing as seen among professionals, thus enhancing the socialization into this text culture (Knain, 2015; Lemke, 1998). Through the writing practices demonstrated by the students, and their reflections about the purposes and functions of writing, the students show confidence that writing can help when struggling with a challenging question. They also express that the competence they acquire through their writing will benefit them in their adult careers, suggesting an awareness of the epistemological functions of writing.

In our study, the students’ conception of the relevance of science lessons during their research process in general, and the writing process in particular, is in contrast to the findings of other studies indicating that science is seen as irrelevant by students (Lyons, 2006). Moreover, the work students undertake is associated with managing situations outside of school as future adults, because according to the students, careers as adults require one to take initiative and produce texts using one's own knowledge. Hence, the experience of acting as researchers, including writing practices in science, was seen as significant by the students, especially outside the school setting (Lemke, 1990; Moje et al., 2001; Norris & Phillips, 2003). A professional awareness is awakened in the students through writing, as they perceive that writing is a crucial aspect of many professions (Klein & Boscolo, 2016). This matches the emphasis on literacy teaching within science (Howes et al., 2009; Sørvik & Mork, 2015). These findings are opposed to studies that have found science literacy in education to be reproductive in nature (Danielsson, 2010; Osborne, 2007).

The students’ research process explored in the present study resembles inquiry described as “a stance that promotes authentic, intentional, and systematic learning” (Mills et al., 2014, p. 36). The work was authentic because the students wanted to find out how far a dog can smell a treat, and they researched it as well as they could. Furthermore, all their actions, both in the research process and in their writing practices, were intentional, serving clear purposes understood by the students: they wanted answers and they wanted to produce a report. Through their research, the students became producers of knowledge. Hence, for them, the boundaries of science moved beyond authoritative texts and predetermined questions from the teacher (Howes et al., 2009, p. 212).

In this study, students acting as researchers create a context in which student initiative and persistence is pivotal to the outcome. These are important facets of being a scientist, together with understanding the process of basic inquiry. Further, the context seems motivating through ownership of the work, doing scientific inquiry to test hypotheses, and communicating with experts, features that have also been highlighted as highly educative by an inquiry competition in Australia (Hubber et al., 2010).

It would be tempting to recommend that all teachers include the kinds of methods described in this study in their science classrooms. However, this might be easier said than done. Working with students as researchers can be time consuming, especially if the students are given the chance to fail through bad planning, deal with messy data, and present new hypotheses along the way. Inquiry demands time, for scientists as well, but time within school must be well utilized. A full‐scale research project within a class can dominate other curricular subjects for a while, and this might not be either desirable or possible in a tight schedule. The educational value of spending 26 hours on investigating the olfactory sense of dogs in elementary school (without obtaining any scientifically valid new knowledge) can be questioned, and to foreground the particular theme in this approach is misleading. The theme is primarily motivating for the students, but the main learning potential lies in the research process and the writing experiences that the students engage in and establish. This shift in viewpoint might be challenging, for both teachers and students, because most schools and teachers tend to be oriented toward content rather than processes, which also explains the fact that reading and writing are often used as replacements for students’ involvement in firsthand investigations (Cervetti et al., 2012).

A classroom with students as researchers requires a teacher who can facilitate the research process. This is even more demanding when the research question at stake is far from the teacher's field of expertise, which can be the case when students are required to find their own research questions. A teacher dedicated to the method, as Howes et al. (2009) emphasized, is therefore crucial. Open inquiry can also fail owing to minimal guidance from the teacher (Kirschner et al., 2006), leaving the students on their own. Furthermore, the learning outcomes for the students might concern the teacher if the local curriculum and other regulating directions narrowly describe expectations for learning. Participating in multiple writing practices, which we assume the students will experience, can enhance learning in the long term but perhaps not for the next test. What is learned through open inquiry might further not be clear (Hattie, 2009) or satisfactory within science (Jiang & McComas, 2015; Minner et al., 2010). Additionally, the teacher's role might be even more crucial in this kind of work (Howes et al., 2009), meaning the results can deviate considerably more in inquiry settings than in traditional settings, where the teacher presents facts.

The students in this study were capable of engaging in relevant writing practices in their research process because they were given opportunity to do so. Thus, agency comes into play. Yet, in addition to opportunity and agency, the methods in this study require that students have a basic understanding of the different ways of using texts. The students collaborated in writing, revising, and discussing texts to function in their setting. It may not always be the case that students are willing or able to work this extensively with texts. The writing of the students in this study is the result of years of training. Additionally, the class was familiar with working as researchers and with the expectations of a written report because this approach had been foregrounded in their previous 6 years of schooling.

## 10 CONCLUSION

We argue that there is a fundamental difference between needing to write on your own account and writing because you are told to. For instance, there is a difference between performing an experiment because you need to write a lab report and writing a report because you want to document an experiment you consider important. Often, this is portrayed as a dichotomy between writing outside and inside of school or a dichotomy between vernacular and dominant literacy (Barton, 2007; Barton & Hamilton, 1998; Street, 1995). In this study, we aimed not to reassert such distinctions but to investigate what characterizes the writing practices of students when they are given the possibility to work collaboratively on a self‐defined research report. This context, of students as researchers, provides socially meaningful ways of using text within the school. Their writing practices are characterized by what they do as aspiring researchers, not as students reproducing textbook content.

When discussing how science texts are read, Howes et al. (2009) stated that there is a principal difference between reading about animals to practice reading skills and reading about animals to find out more about a question asked based on observations. Only the latter situations place literacy in the service of science inquiry, which was the case among the observed students in the present study. School literacy is often narrow, and the writing events students take part in during lessons in school science often have little relevance for practices outside of school. Allowing students to act as researchers and confront the struggles of the research process provides them with valuable experience of varied literacy practices and approximates the way in which adult scientists work. Through careful facilitation of the research process by the teacher, the students in our study have taken part in the scientific text culture, using texts and writing for purposes beyond those traditionally associated with school science. While the research process these students undertook did not result in any new scientifically valid knowledge about dog's olfaction, we see evidence that the literacy practices they engaged in were considered meaningful in the here‐and‐now situation. In addition, the students experienced these ways of writing as more authentic and relevant for their future as professional adults.

In our fast‐changing world, learning to ask questions, gather information, and present findings and ideas will always be relevant, and students should experience these ways of acting in the world of texts as early as possible.

## ENDNOTES

1 Some researchers refer to NLS simply as “literacy studies” because the term has been used for almost 30 years. For a discussion of the terms (new) literacy studies and new literacies, we refer to Gillen and Merchant (2013).

2 “Learning to write” and “Writing to learn” correspond to the dichotomy between writing in the disciplines and writing across the curriculum, e.g., Carter (2007).

3 We are grateful to Kirsti Klette and Torgeir Christiansen at the Department of Teacher Education and School Research, University of Oslo, for lending us cameras.

4
http://www.qsrinternational.com/what-is-nvivo.
